# Intranasal dexmedetomidine for procedural sedation in children: a systematic review and meta-analysis

**DOI:** 10.1016/j.bjane.2025.844717

**Published:** 2025-12-05

**Authors:** Kelvin Oliveira Rocha, Ellen Renata Ferreira de Araújo Santos, Mariana Madureira Pombeiro, Márcia Nayara da Silva Leite Fidelis, Fabiana Maria Kakehasi, Daniela Caldas Teixeira

**Affiliations:** a Universidade Federal de Minas Gerais (UFMG), Hospital das Clínicas, Belo Horizonte, MG, Brazil; b Universidade Federal de Minas Gerais (UFMG), Hospital das Clínicas, Departamento de Pediatria, Belo Horizonte, MG, Brazil

**Keywords:** Dexmedetomidine, Meta-Analysis, Pediatrics, Procedural sedation

## Abstract

**Background:**

Intranasal Dexmedetomidine (IN-DEX) is a promising agent for pediatric procedural sedation due to its non-invasive route and favorable safety profile. However, a comprehensive synthesis quantifying its clinical timeline and safety as monotherapy is lacking. This meta-analysis assesses the efficacy and adverse events of IN-DEX as a standalone sedative in children.

**Methods:**

Following PRISMA 2020 guidelines and PROSPERO registration (CRD420250652456), this meta-analysis systematically searched PubMed, ScienceDirect, and SciELO for intranasal dexmedetomidine monotherapy in children under 18 years from January 1, 2003, to July 1, 2025. Key outcomes included sedation success, onset, and duration. Data were pooled using a random-effects model, with risk-of-bias assessed via RoB2. We performed sensitivity and subgroup analyses and evaluated evidence certainty using the GRADE approach.

**Results:**

Twenty-eight RCTs were included. The overall pooled mean onset time was 18.9 minutes and duration was 60.3 minutes, though both had very low evidence certainty due to high heterogeneity (I² > 99%). The overall success rate was 79.58%. Notably, in a subgroup of low-to-moderate risk-of-bias studies, a dose of [2, 3) mcg.kg^-1^ achieved an 84.04% success rate, supported by high-quality evidence (GRADE: High, I² = 0%). The pooled proportions for key adverse events were hypotension (8.24%), bradycardia (5.08%), and desaturation (2.76%).

**Conclusion:**

IN-DEX is an effective monotherapy for pediatric procedural sedation. Doses of [2, 3) mcg.kg^-1^ are associated with high success rates, supported by high-quality evidence. While IN-DEX demonstrates a favorable respiratory profile with low desaturation rates, its use requires vigilant hemodynamic monitoring due to the risks of hypotension and bradycardia.

## Introduction

Procedural sedation and premedication in the pediatric population represent a significant clinical challenge, requiring strategies that minimize anxiety and distress while ensuring patient safety and cooperation.^1^ The ideal sedative should be effective, have a favorable safety profile with minimal respiratory depression, and be administered through a non-invasive route to avoid further distress.^2^ In this context, needle-free options are particularly valuable.

Dexmedetomidine, a highly selective alpha-2 adrenergic agonist, has emerged as a promising agent for procedural sedation in ambulatory and emergency settings. Its pharmacological properties, providing anxiolysis, sedation, and analgesia without significant respiratory compromise, make it an attractive alternative to traditional sedatives.^3^^,^^4^ Although its use for this indication in children is largely off-label, its Intranasal (IN) administration has gained popularity due to its ease of use and rapid systemic absorption through the nasal mucosa.^5^

The use of dexmedetomidine in the pediatric population has been the subject of several systematic reviews, although their focus has often been on specific clinical scenarios other than procedural sedation. For instance, meta-analyses have investigated its role in preventing perioperative respiratory adverse events during general anesthesia or have focused on direct comparisons against oral midazolam for the purpose of premedication.^6^^,^^7^ While this body of work is valuable, a significant gap remains regarding the use of Intranasal Dexmedetomidine (IN-DEX) as a standalone sedative agent for procedural sedation. Specifically, a comprehensive meta-analysis that provides pooled, quantitative estimates for key clinical parameters, such as sedation onset time and duration of action, is currently lacking. Furthermore, prior reviews have not centered on quantifying the pooled incidence rates of key adverse events across a broad spectrum of pediatric procedures. Therefore, an updated synthesis focusing on IN-DEX as monotherapy is needed to provide clinicians with robust data on its clinical timeline and safety profile, stratified by dose and procedure type.

While prior reviews are valuable, they often focus on comparative efficacy (e.g., IN-DEX vs. other drugs) or its use in combination with other agents.^7^ In contrast, a quantitative synthesis focused strictly on IN-DEX monotherapy, providing robust estimates of its intrinsic clinical variables (like onset time, duration, and safety) to aid clinical planning, is lacking. To our knowledge, this is the first review to address this gap and stratify these key outcomes by dose range to identify an optimal therapeutic window.

This study aims to assess the efficacy and adverse events associated with IN-DEX in pediatric patients, considering dose stratification and types of procedures.

## Methods

### Protocol and registration

This systematic review and meta-analysis was conducted and reported in accordance with the 2020 PRISMA (Preferred Reporting Items for Systematic Reviews and Meta-Analyses) guidelines. The completed PRISMA checklist is provided in Appendix A. The study protocol was prospectively registered in the International Prospective Register of Systematic Reviews (PROSPERO)^8^ and is available in ID CRD420250652456.

### Data sources and search strategy

**Figure 1:**
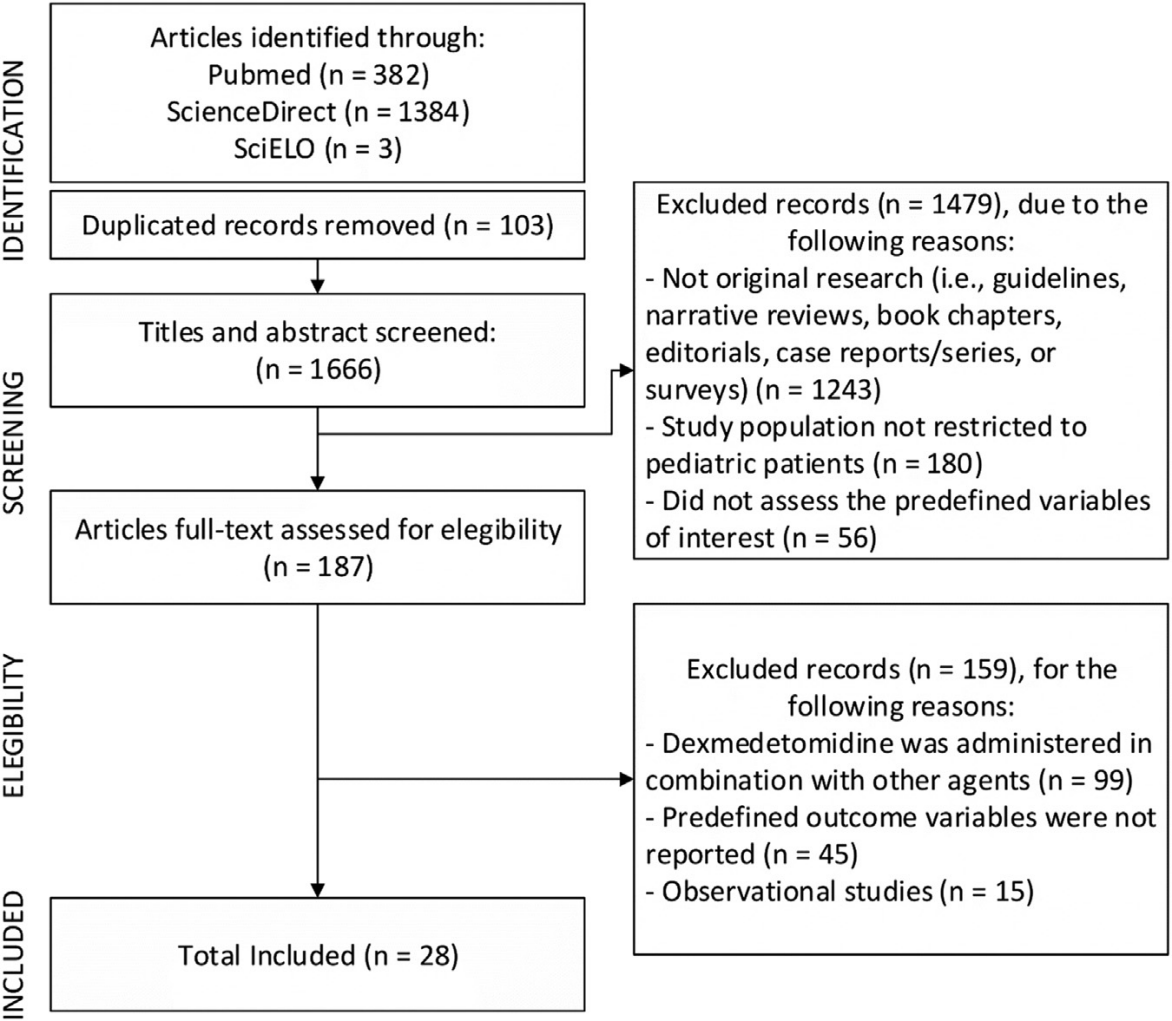
PRISMA flow diagram illustrating the study selection process.

A systematic search was conducted on PubMed, ScienceDirect, and SciELO, covering the period from January 1, 2003, to July 1, 2025. ScienceDirect was utilized as a search database for content hosted on its platform, in addition to its use for full-text retrieval (Fig. 1). The full, database-specific search strategies are provided in Appendix A. No language restrictions were applied.

### Eligibility criteria

**Figure 2:**
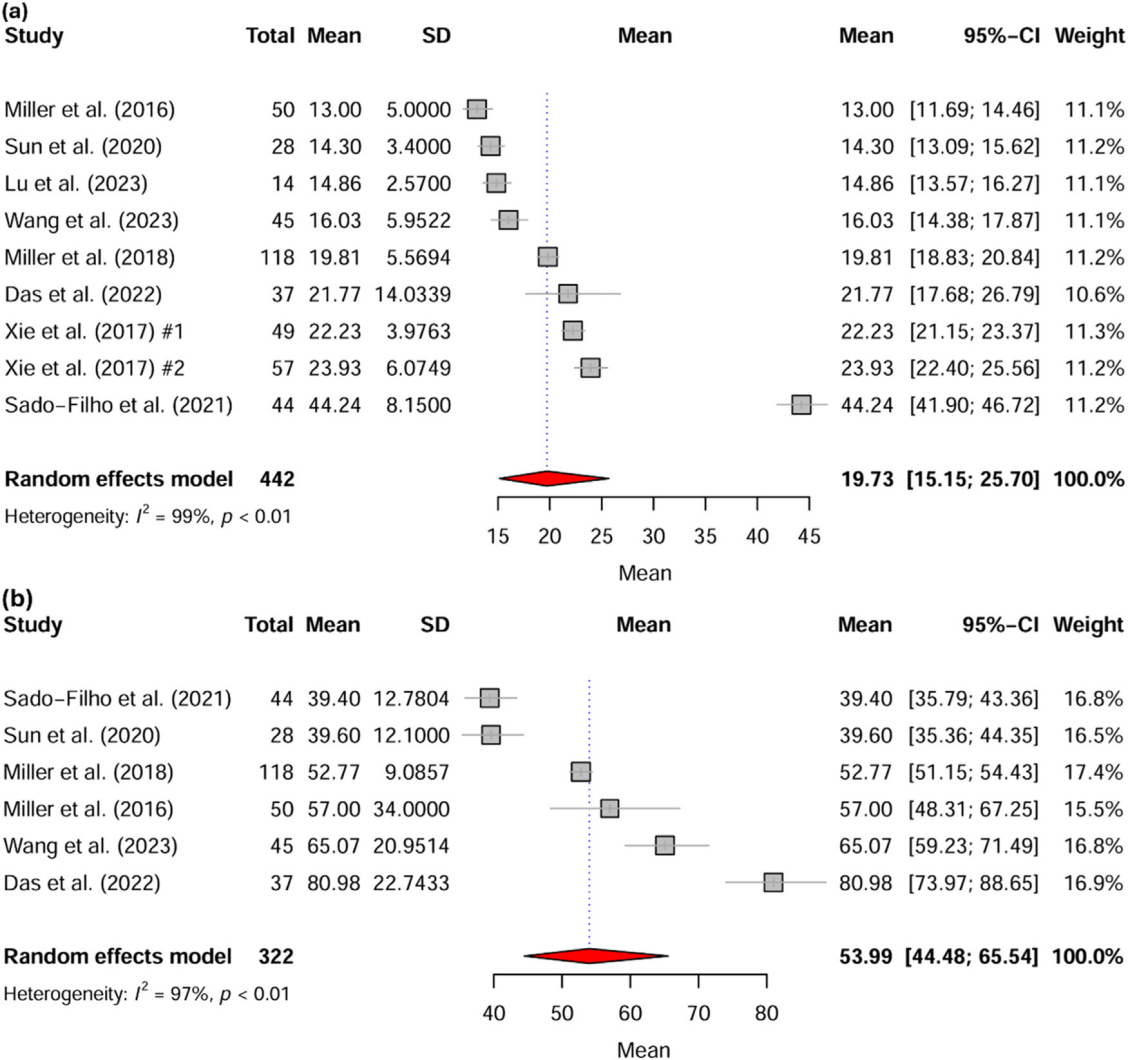
Forest plot of the weighted mean (a) onset time and (b) duration of IN-DEX at [2, 3) mcg.kg^-1^/dose in pediatric patients, excluding studies rated as high-risk studies according to the RoB2 tool. Xie et al. (2017) presents two distinct groups, #1 with mucosal atomization device, #2 with serynge device.

**Figure 3:**
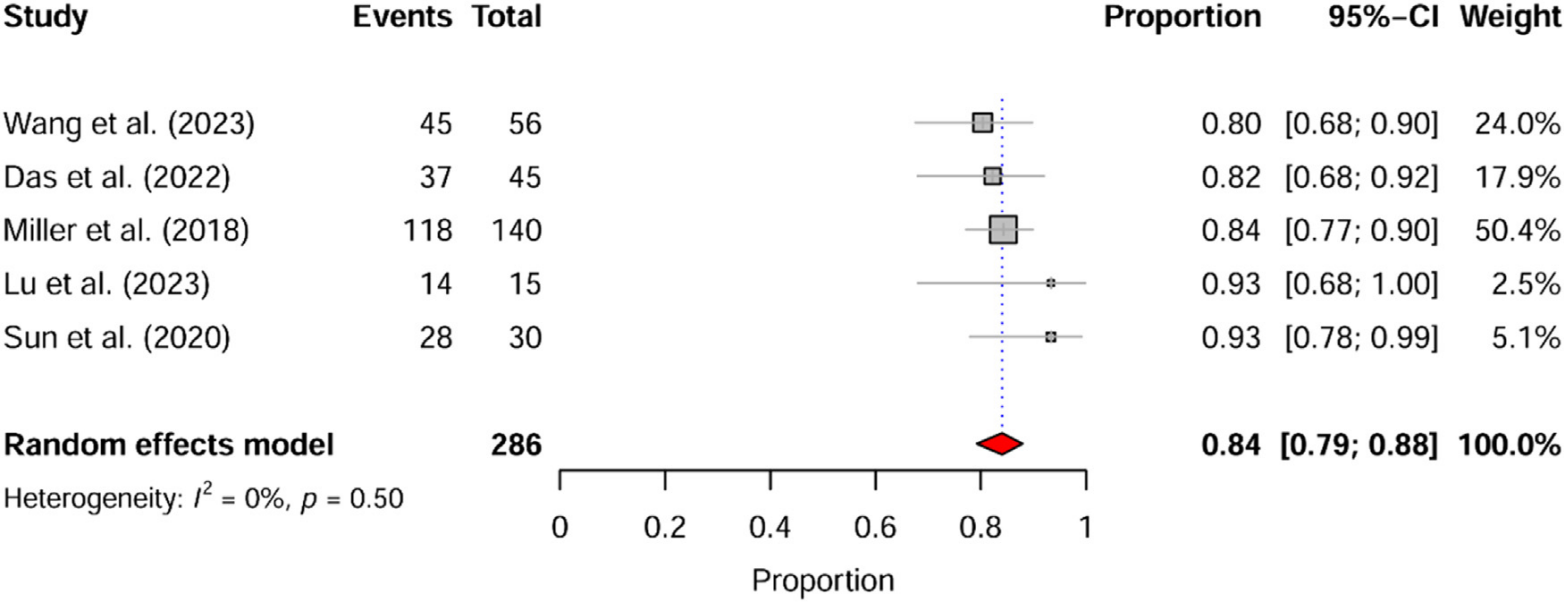
Forest plot of the weighted success proportion in pediatric patients receiving IN-DEX at (2, 3] mcg.kg^-1^/dose, excluding studies rated as high-risk studies according to the RoB2 tool.

Studies were included if they met the following criteria: a) Study design: randomized clinical trial; b) Population: patients under 18 years of age; c) Intervention: at least one group receiving IN-DEX as monotherapy; d) Outcomes: studies reporting central tendency measures for at least one of the following outcomes ‒ time to sedation onset, duration of sedation, or sedation success rate. Exclusion criteria were: a) Study design: reviews, observational studies, case reports, case series, letters to the editor; b) Population: studies including participants older than 18 years; c) Intervention: studies that did not include at least one group receiving IN-DEX as monotherapy; d) Outcomes: studies lacking primary outcome variables or failing to report measures of central tendency. In line with research integrity policies, all included studies were screened for retractions, expressions of concern, or serious methodological/ethical flags using the Retraction Watch and PubPeer databases (Figure 2, Figure 3).

### Study selection

Study selection was performed in two phases by independent reviewers (KOR, MMP). Titles and abstracts were manually screened for relevance, followed by full-text assessment of potentially eligible articles. Disagreements were resolved by a third reviewer (ERFAS).

### Data extraction

Two reviewers (KOR, MMP) independently and manually extracted relevant data using a standardized Microsoft Excel 365 spreadsheet. Extracted information included study characteristics, patient demographics, intervention details, and outcome data. For multi-arm trials that included both monotherapy and combination therapy groups, only data from the IN-DEX monotherapy arm and its relevant comparator arm (e.g., placebo, another active drug) were extracted for inclusion in this meta-analysis.

### Data synthesis and statistical analysis

Medians and interquartile ranges were converted to means and standard deviations using the Box-Cox method as described by McGrath et al. (2020), as this approach is more appropriate for non-parametric data distributions.^9^ Age-related central tendency measures were normalized to a yearly scale. Weighted mean prevalence, overall mean and 95% Confidence Intervals (95% CIs) and 95% Prediction Intervals (PIs) were calculated using a random-effects model with the DerSimonian and Laird estimator, applying logit transformation to stabilize distributions.

Study heterogeneity was assessed using the I^2^ statistic, which estimates the proportion of variability not attributable to sampling error. Heterogeneity was considered substantial when I^2^ exceeded 50%. Publication bias was evaluated both subjectively through funnel plot inspection and objectively using Egger’s test (Appendix A). Egger’s test was applied only to outcomes reported in ten or more studies. All statistical analyses were performed in R using the meta package (version 7.0-0), with a type I error threshold of 5%.

## Definitions

### Success and adverse events

Success was defined as completing the procedure without the need for additional sedatives or repeated sedation dosing. Definitions for hypotension, bradycardia, and desaturation from each article were extracted and are presented in Appendix A.

### Invasiveness categories

Procedures were classified into four levels of invasiveness based on the degree of physical contact, tissue penetration, and pain potential: (0) Non-invasive and painless: no direct contact with tissues or induction of pain (e.g., imaging exams, electroencephalogram, electrocardiogram); (1) Minimally invasive: light contact with minimal discomfort, without significant tissue penetration (e.g., ophthalmologic examinations); (2) Moderately invasive: superficial penetration with mild to moderate pain (e.g., venous cannulation); (3) Invasive with pain potential: deep tissue manipulation or procedures associated with significant pain (e.g., dental procedures).

### Age strata

Studies were classified into pediatric strata based on the mean age of participants: Neonatal (0‒28 days), Infant (0‒1 year), Toddler (1‒3 years), Preschooler (3‒6 years), Child (6‒12 years), and Adolescent (12‒19 years). When the mean age clearly aligned with a specific stratum, that category was adopted. In cases of broad age ranges, the standard deviation was considered to identify the predominant concentration.

### Assessment of risk of bias in included studies and risk of publication bias

Two authors (KOR, MMP) independently assessed the quality and risk of bias of the included studies using the Cochrane Risk of Bias 2.0 (RoB2) tool for randomized controlled trials (Appendix A).^10^^,^^11^

### Sensitivity assessment and subgroup analysis

To assess the robustness of the primary outcome, pre-specified sensitivity analyses were conducted by repeating the main analysis while sequentially excluding studies with a high risk of bias and studies conducted in China. The results were considered robust if the direction and statistical significance of the pooled effect estimate did not change substantially.

### Quality of evidence

Evidence certainty for each primary outcome was independently assessed by two authors using the GRADE approach. Starting from a 'high' rating for randomized trials, certainty was downgraded based on five domains: risk of bias, inconsistency, indirectness, imprecision, and publication bias. Final ratings were classified as high, moderate, low, or very low.

## Results

### Descriptive analysis

In the 28 clinical trials analyzed (Table 1), a geographical concentration of studies in Asia was observed, with the majority being conducted in China (*κ* = 12; n = 922) and India (κ = 8; n = 249), followed by the United States (κ = 3; n = 207). The pediatric population in these trials was mainly composed of preschoolers (κ = 10; n = 503) and toddlers (κ = 9; n = 678). Regarding the clinical context, IN-DEX was predominantly used for non-invasive and painless procedures (κ = 13; n = 921) but was also applied in scenarios with potential for pain (κ = 8; n = 338), moderately invasive (κ = 5; n = 209), and minimally invasive procedures (κ = 2; n = 161). The dosage stratification for these trials revealed a predominance of doses in the [2, 3) mcg.kg^-1^ range (κ = 17; n = 830). Higher doses, ≥ 3 mcg.kg^-1^, were also frequently investigated (κ = 9; n = 668), while lower doses in the [1, 2) mcg.kg^-1^ range were less common (κ = 4; n = 131).

**Table 1:** Overview of pediatric studies using intranasal dexmedetomidine.

Author (year, country)	Description of the study population	DEX doses	Device	Sedation scale ‒ criteria for success	Definitions for adverse events	Risk of bias
Xie et al. (2017, China)[11]	106 ASA I–II children for eye surgery; evaluated sedation via spray or syringe drops	2 mcg.kg^-1^	Atomizer and Drops by Syringe	FLACC ‒ Conclusion of the procedure.	SpO_2_ < 94%	Low
Wang et al. (2024, China)[12]	105 infants with cleft lip/palate for CT scan; compared dexmedetomidine alone vs. combo with midazolam	2 mcg.kg^-1^	Atomizer	RSS ‒ Conclusion of the procedure.	HR < 60 bpm, SpO_2_< 90%	Low
Li et al. (2019, China)[13]	275 ASA I–II autistic children for CT/ABR; received intranasal dexmedetomidine alone or with buccal midazolam	3 mcg.kg^-1^	Not specified	UMSS ‒ Conclusion of the procedure.	HR < 20% from baseline, SBP < 20% from baseline, SpO_2_ < 94%	Moderate
Chandrasekar et al. (2023, India)[14]	195 ASA I–II children scheduled for MRI; compared triclofos, midazolam, and dexmedetomidine sedation	3 mcg.kg^-1^	Not specified	PSSS ≤ 3	SBP < 20% from baseline, SpO_2_ < 92%	High
Qiao et al. (2017, China)[15]	135 ASA I–II children (2–6 yrs) for eye surgery; received dexmedetomidine, ketamine, or both	2.5 mcg.kg^-1^	Not specified	Sedation Scale (SS-5) ‒ Conclusion of the procedure.		High
Patel et al. (2018, India)[16]	44 ASA I uncooperative children (4–9 yrs) for dental care; compared dexmedetomidine doses/routes	2 to 2.5 mcg.kg^-1^	Not specified	SS-5 ≥ 3 and SpO_2_ ≥ 90%		High
Ibrahim et al. (2014, Egypt)[17]	63 ASA I–II children (4–10 yrs) for MRI; compared intranasal dexmedetomidine and ketamine + IV midazolam	3 mcg.kg^-1^	Not specified	RSS ‒ Conclusion of the procedure.	HR < 20% from baseline, SBP < 20% from baseline, SpO^2^ < 92%	High
Panda et al. (2021, India)[18]	100 ASA I–II children (< 3 yrs) for echocardiography; compared intranasal dexmedetomidine vs. midazolam	2 mcg.kg^-1^	Drops by Syringe	RSS ≥ 3	HR < 20% from baseline, SpO_2_ < 92%	High
Yuen et al. (2012, China)[1]	116 ASA I–II children (7–17 kg) for elective surgery; grouped by age and hospital	1 mcg.kg^-1^ and 2 mcg.kg^-1^	Drops by Syringe	SS-5 ≥ 3		High
Li et al. (2016, China)[19]	279 ASA I–III children for echocardiography; received dexmedetomidine via atomizer or drops	3 mcg.kg^-1^	Atomizer and Drops by Syringe	UMSS ≥ 2	HR < 20% from baseline, SBP < 20% from baseline, SpO_2_ < 92%	Low
Yuen et al. (2017, China)[20]	196 ASA I–II children for CT scan; received oral chloral hydrate or intranasal dexmedetomidine	3 mcg.kg^-1^	Atomizer	UMSS ≥ 2	HR < 20% from baseline, SpO_2_ < 94%	Low
Janiani et al. (2024, India)[21]	15 ASA I children with negative dental behavior; underwent molar pulpectomy	1 mcg.kg^-1^	Atomizer	RSS ‒ Conclusion of the procedure.	SpO_2_ < 94%	High
Azizkhani et al. (2020, Iran)[22]	162 children in emergency room for CT; randomized to Dexmedetomidine or midazolam sedation	3 mcg.kg^-1^	Atomizer	RSS ≥ 3		High
Gupta et al. (2017, India)[23]	60 ASA I–II children for elective brain MRI; received midazolam or dexmedetomidine	1 mcg.kg^-1^	Drops by Syringe	MOAA/S ≤ 3	SpO_2_ < 94%	High
Miller et al. (2018, USA)[24]	280 infants (3–24 months) with heart disease for echocardiography; received dexmedetomidine or oral pentobarbital	2.5 mcg.kg^-1^	Atomizer	RSS > 3	HR < 80 bpm, SBP < 70, SpO_2_ < 92%	Low
Qian et al. (2020, China)[25]	63 ASA I–II children (3–7 yrs) for tonsillectomy; received intranasal dexmedetomidine or dexmedetomidine + ketamine	2 mcg.kg^-1^	Not specified	MOAA/S ≤ 3		High
Das et al. (2022, India)[26]	90 ASA I–III children (3–6 yrs) with cancer; 21 radiotherapy sessions with dexmedetomidine or oral midazolam + ketamine	2 mcg.kg^-1^	Not specified	RSS = 3	SBP < 20% from baseline	Low
Sado-Filho et al. (2021, Brazil)[27]	88 ASA I–II children (1–7 yrs) with poor dental behavior; treated with dexmedetomidine or dexmedetomidine + ketamine	2.5 mcg.kg^-1^	Not specified	OSUBRS > 50%	SpO_2_ < 88%	Low
Surendar et al. (2014, India)[28]	84 ASA I children (4–14 yrs) uncooperative in dental care; received one of four intranasal sedation protocols	1 mcg.kg^-1^ and 1.5 mcg.kg^-1^	Not specified	SRSB > 2 and SpO_2_ > 90%	SBP < 20% from baseline, SpO_2_ < 90%	High
Chen et al. (2019, China)[29]	100 ASA I–II children with congenital cataracts for ophthalmologic exams	2 mcg.kg^-1^ and 3 mcg.kg^-1^	Not specified	MOAA/S ≤ 3	SpO_2_ < 94%	High
Lu et al. (2023, China)[30]	40 hospitalized burn patients (5–45 months); received dexmedetomidine drops or chloral hydrate enema for Peripherally Inserted Central Catheter	2 mcg.kg^-1^	Drops by Syringe	RSS ≥ 3		Low
Miller et al. (2016, USA)[31]	150 infants (3–36 months) with heart disease; received dexmedetomidine 2 µg.kg^-1^, 3 µg.kg^-1^, or oral chloral hydrate	2 mcg.kg^-1^	Drops by Syringe	RSS ≥ 3	HR < 80 bpm, SpO_2_ < 92%	Moderate
Ghai et al. (2017, India)[32]	59 ASA I–II children (1–6 yrs) for CT; received oral midazolam or intranasal dexmedetomidine 2.5 µg.kg^-1^	2.5 mcg.kg^-1^	Drops by Syringe	RSS ≥ 4		High
Reynolds et al. (2016, USA)[33]	85 children (6 months–8 yrs, 5–25 kg) for ABR; excluded prior sedation failure and comorbidities	3 mcg.kg^-1^	Not specified	Own adapted scale ‒ Conclusion of the procedure.	SpO_2_ < 90%	Moderate
Cao et al. (2017, China) [34]	141 ASA I–II children (3–36 months) with cataracts; received intranasal dexmedetomidine or oral chloral hydrate	2 mcg.kg^-1^	Not specified	RSS ‒ Conclusion of the procedure.	HR < 60 bpm, SpO_2_ < 94%	High
Sun et al. (2020, China) [35]	60 ASA I–II infants (1–36 months) with heart disease; received dexmedetomidine alone or dexmedetomidine + ketamine	2 mcg.kg^-1^	Drops by Syringe	MOAA/S ≤ 3	SpO_2_ < 90%	Low
Nikula et al. (2024, Sweden) [3]	148 healthy Swedish children (3–15 yrs) with fractures or burns < 4%; treated in emergency room	2 mcg.kg^-1^	Drops by Syringe	RSS ≥ 2	HR < 20% from baseline, SpO_2_ < 94%	High
Tug et al. (2015, Turkey) [36]	60 ASA I–II healthy children (1–10 yrs) for MRI; received 3 or 4 µg.kg^-1^ dexmedetomidine with recovery and side effect tracking	3 mcg.kg^-1^ to 4 mcg.kg^-1^	Not specified	RSS = 5	HR < 60 bpm, SpO_2_ < 94%	Moderate

ASA, American Society of Anesthesiologists; CT, Computed Tomography; EEG, Electroencephalogram; MRI, Magnetic Resonance Imaging; HR, Heart Rate; SBP, Systolic Blood Pressure; FLACC, Face, Legs, Activity, Cry, Consolability Scale; RSS, Ramsay Sedation Scale; OSUBRS, Ohio State University Behavioral Rating Scale; SRSB, Sedation Rating Scale for Behavior; MOAAS, Modified Observer’s Assessment of Alertness/Sedation Scale; UMSS, University of Michigan Sedation Scale; PSSS, Pediatric Sedation State Scale.

For studies with multiple intervention arms, only data from the intranasal dexmedetomidine monotherapy group were extracted for this analysis.

### Inferential assessment

#### Onset time

The analysis for sedation onset time included 34 distinct groups with 1,609 participants. The overall pooled mean onset time was 18.9 minutes (95% CI: 16.6‒21.4; 95% PI: 8.7‒41.1; I^2^ = 99%; GRADE: Very low). A sensitivity analysis restricted to 16 distinct groups (897 participants) with low or moderate risk of bias yielded a mean onset time of 20.5 minutes (95% CI: 17.3‒24.3; 95% PI: 9.7‒43.5; I^2^ = 97.5%; GRADE: Low). A further sensitivity analysis did not improve the heterogeneity, as shown in Table 2. Excluding Chinese clinical trials, which included 18 study groups (687 participants), we found a mean onset time of 18.9 minutes (95% CI: 15.0‒23.8); I^2^ = 99.5%; GRADE: Very low). In a meta-regression analysis restricted to studies with low or moderate risk of bias, both procedural invasiveness (p = 0.009) and IN-DEX dosage (p = 0.01) were significantly associated with an increase in sedation onset time, as shown in Appendix A.

**Table 2:** Sensitivity assessment of onset time across clinical trial publications – restricted to studies without high risk of bias according to RoB2.

Onset Time					
Variables	K (Events / N)	Mean (95% CI) [95% PI]	I^2^	Ajusted Mean (95% CI)	GRADE
Onset Time General (high-risk included)	34 (34/1609)	18.9 (16.6 ‒ 21.4) [8.7 ‒ 41.1]	99.3	15.5 (13.6 ‒ 17.8)	Very low
High-risk of bias excluded	16 (16/897)	20.5 (17.3 ‒ 24.3) [9.7 ‒ 43.5]	98.6	22.4 (19.0 ‒ 26.4)	Low
*Infant*	2 (2/146)	16.9 (12.3 ‒ 23.2) [‒]	97.4	‒	Very low
*Toddler*	7 (7/489)	17.4 (15.2 ‒ 20.0) [10.6 ‒ 28.7]	95.3	17.4 (15.2 ‒ 20.0)	Low
*Preschooler*	6 (6/217)	28.0 (20.8 ‒ 37.5) [9.5 ‒ 82.6]	98.6	29.2 (22.3 ‒ 38.3)	Very low
*Non-invasive and painless*	12 (12/733)	19.2 (17.1 ‒ 21.5) [12.1 ‒ 30.3]	95.3	18.6 (16.5 ‒ 20.8)	Low
*Moderately invasive*	2 (2/106)	23.0 (21.4 ‒ 24.7) [‒]	67.1	**‒**	Low
*Invasive with potential for pain*	2 (2/58)	25.7 (8.8 ‒ 74.7) [‒]	99.7	‒	Very low
*Dose: [2, 3) mcg.kg*^*-1*^	9 (9/442)	19.7 (15.1 ‒ 25.7) [7.3 ‒ 53.7]	99.1	24.2 (18.8 ‒ 31.2)	Very low
*Dose: ≥ 3 mcg.kg*^*-1*^	7 (7/455)	21.4 (18.2 ‒ 25.0) [12.1 ‒ 37.7]	95.7	18.8 (15.9 ‒ 22.2)	Low

**Duration**					

**Variables**	**K (Events / N)**	**Mean (95% CI) [95% PI]**	**I^2^**	**Ajusted Mean (95% CI)**	**GRADE**

Duration time general (high-risk included)	28 (28/1368)	60.3 (52.7 ‒ 69.1) [28.3 ‒ 128.4]	99.3	60.3 (52.7 ‒ 69.1)	Low
High-risk of bias excluded	11 (11/674)	54.6 (47.8 ‒ 62.4) [32.6 ‒ 91.6]	97.2	49.0 (42.6 ‒ 56.2)	Low
*Infant*	2 (2/146)	46.0 (34.7 ‒ 60.9) [‒]	95.6	‒	Very low
*Toddler*	4 (4/372)	51.0 (40.9 ‒ 63.6) [17.7 ‒ 147.4]	97.3	44.8 (34.6 ‒ 58.0)	Very low
*Preschooler*	4 (4/111)	61.9 (42.8 ‒ 89.7) [10.4 ‒ 369.6]	97.5	49.3 (33.7 ‒ 72.1)	Very low
*Non-invasive and painless*	10 (10/630)	56.5 (49.1 ‒ 64.9) [33.4 ‒ 95.6]	97.2	50.0 (43.4 ‒ 57.7)	Very low
*Dose: [2, 3) mcg.kg*^*-1*^	6 (6/322)	54.0 (44.5 ‒ 65.5) [26.5 ‒ 110.0]	96.9	54.0 (44.5 ‒ 65.5)	Low
*Dose: ≥ C3 mcg.kg*^*-1*^	5 (5/352)	55.5 (44.6 ‒ 69.1) [23.9 ‒ 129.2]	97.4	45.9 (36.6 ‒ 57.5)	Very low

**Success Rates**					

**Variables**	**K (Events / N)**	**Proportion (95% CI) [95% PI]**	**I^2^**	**Ajusted Proportion**	**GRADE**

Success general (high-risk included)	17 (897 / 1132)	79.58% (73.56 ‒ 84.52) [51.17 ‒ 93.55]	77.33	76.54% (70.21 ‒ 81.88)	Low
High-risk of bias excluded	12 (697 / 893)	78.23% (70.31 ‒ 84.51) [45.08 ‒ 94.02]	81.4	75.02% (66.81 ‒ 81.76)	Very low
*Infant*	2 (146 / 170)	87.08% (75.16 ‒ 93.76) [‒]	35.94	‒	Low
*Toddler*	6 (439 / 562)	79.96% (71.02 ‒ 86.66) [45.69 ‒ 94.98]	77.97	76.64% (67.22 ‒ 83.99)	Very low
*Preschooler*	3 (67 / 105)	62.53% (29.44 ‒ 86.97) [0.00 ‒ 100.00]	89.57	62.53% (29.44 ‒ 86.97)	Very low
*Non-invasive and painless procedure*	11 (683 / 878)	77.48% (69.29 ‒ 83.99) [43.58 ‒ 93.87]	82.52	74.98% (66.58 ‒ 81.84)	Very low
*Dose: [2, 3) mcg.kg*^*-1*^	5 (242 / 286)	84.04% (79.21 ‒ 87.91) [75.70 ‒ 89.90]	0	83.30% (77.95 ‒ 87.57)	**High**
*Dose: ≥ 3 mcg.kg*^*-1*^	7 (455 / 607)	73.05% (60.78 ‒ 82.58) [28.60 ‒ 94.83]	86.78	73.05% (60.78 ‒ 82.58)	Very low

All variables following “High-risk of bias excluded” refer specifically to studies classified as having low or moderate risk of bias.

General: overall group encompassing all event subgroups definitions.

K, Distinct Subgroups.

Adjusted proportions represent the estimated values following correction using the *trim and fill* method in groups showing evidence of publication bias.

### Duration

The analysis of sedation duration time included 28 study groups (1,368 participants), yielding a pooled mean duration of 60.3 minutes (95% CI: 52.7‒69.1; 95% PI: 28.3‒128.4; I^2^ = 99.3%; GRADE: Low), as shown in Table 2. Sensitivity analysis restricted to studies with low or moderate risk of bias (11 groups, 674 participants) found a mean duration of 54.6 minutes (95% CI: 47.8‒62.4; 95% PI: 32.6‒91.6; I^2^ = 97.2%; GRADE: Low). A separate sensitivity analysis restricted to non-Chinese clinical trials (16 groups, 630 participants) yielded a similar mean duration of 58.0 minutes (95% CI: 50.4‒66.7; I^2^ = 98.7%; GRADE Very low). A meta-regression, limited to studies not classified as high risk of bias, identified that sedation duration significantly increased with the mean age of participants (expB = 1.06; p = 0.018; I² = 95.5%; R^2^ = 37.2%), while procedural invasiveness (p = 0.118) and dexmedetomidine dose (p = 0.446) were not significant predictors.

### Success

The following analysis of procedural success was restricted to studies defining this outcome as the ability to complete the intervention without administering supplemental sedatives or repeating the initial sedation dose. The overall success rate, evaluated across 17 clinical trial groups (1,132 participants), yielded a pooled proportion of 79.58% (95% CI: 73.56‒84.52; 95% PI: 51.17‒93.55; I^2^ = 77.33%; GRADE: Low). A sensitivity analysis excluding studies with a high risk of bias (12 groups, 893 participants) resulted in a success rate of 78.23% (95% CI: 70.31‒84.51; 95% PI: 45.08‒94.02; I^2^ = 81.4%; GRADE: Low). In the overall analysis, the subgroup with a dose of [2, 3) mcg.kg^-1^ had a success rate of 82.45% (95% CI: 77.46‒86.52; 95% PI: 70.66‒90.16; I^2^ = 25.12%; GRADE Moderate). When restricted to low- and moderate-risk studies, this same dose range demonstrated a success rate of 84.04% (95% CI: 79.21‒87.91; 95% PI: 75.70‒89.90; I^2^ = 0%; GRADE: High). Evidence certainty was rated High as this finding was based on low-risk studies, demonstrated no inconsistency (I^2^ = 0%), and yielded a precise effect estimate. A multivariable meta-regression did not find a significant correlation between the success rate and mean age, procedural invasiveness, dexmedetomidine dose, or RoB2 score (p > 0.05 for all variables).

### Hypotension

The overall proportion of hypotension across 9 study groups (597 participants) was 8.24% (95% CI: 5.06‒13.16; I^2^ = 55.81%; GRADE: very low). A sensitivity analysis excluding studies with a high risk of bias (5 groups, 477 participants) yielded a similar proportion of 8.61% (95% CI: 4.59‒15.57; 95% PI: 1.00‒46.71; I^2^ = 72.07%; GRADE: very low) (Table 3). The highest quality of evidence emerged from this subset of low and moderate-risk studies for hypotension defined as a SBP decrease of 20% from baseline; this event occurred in 6.94% of participants (95% CI: 4.68‒10.20), with no heterogeneity and a moderate quality of evidence (I^2^ = 0%; GRADE: moderate). A primary multivariable meta-regression of the full dataset identified both mean age (p = 0.013) and the Risk of Bias score (RoB2) (p = 0.021) as significant predictors of hypotension. However, in a subsequent meta-regression restricted to studies without a high risk of bias, mean age remained the sole significant predictor (ExpB = 0.298; p = 0.014), with younger age associated with a higher proportion of hypotension. Procedural invasiveness, IN-DEX dose, and the RoB2 score were not significant in this stricter model.

**Table 3:** Evaluation of the weighted proportion of key adverse events associated with IN-DEX, restricted to studies assessed as low or moderate risk of bias by the RoB2 tool.

Variables	K (Events / N)	Proportion (95% CI) [PI 95%]	I^2^	Ajusted Proportion	GRADE
Hypotension general	5 (45 / 477)	8.61% (4.59 ‒ 15.57) [1.00 ‒ 46.71]	72.07	13.80% (7.79 ‒ 23.28)	Very low
*SBP < 20% from baseline*	4 (23 / 359)	6.94% (4.68 ‒ 10.20) [2.89 ‒ 15.79]	0	7.21% (4.88 ‒ 10.52)	**Moderate**
Bradycardia general	9 (31 / 629)	4.78% (1.97 ‒ 11.12) [0.36 ‒ 41.02]	72.57	11.71% (4.87 ‒ 25.55)	Very low
*HR < 20% from baseline*	4 (14 / 386)	1.56% (0.09 ‒ 21.22) [0.00 ‒ 99.98]	86.38	11.83% (1.14 ‒ 60.90)	Very low
Desaturation general	14 (11 / 846)	3.07% (1.90 ‒ 4.92) [1.80 ‒ 5.19]	0	4.02% (2.24 ‒ 7.14)	**Moderate**
*SpO*_*2*_*< 92%*	4 (9 / 401)	2.98% (1.01 ‒ 8.45) [0.05 ‒ 64.27]	50.18	3.89% (1.24 ‒ 11.59)	Very low
*SpO*_*2*_*< 90%*	3 (2 / 112)	3.28% (1.06 ‒ 9.72) [0.00 ‒ 98.37]	0	5.13% (1.96 ‒ 12.77)	Very low

CI, Confidence Interval; PI, Prediction Interval; General, Overall group including all event definitions; K, Distinct subgroups; SBP, Systolic Blood Pressure; HR, Heart Rate.

Adjusted proportions represent estimates corrected using the trim-and-fill method in groups with evidence of publication bias.

### Bradycardia

The overall proportion of bradycardia across 13 study groups (839 participants) was 5.08% (95% CI: 2.61‒9.67; 95% PI: 0.36‒41.02), with a high degree of heterogeneity and a very low quality of evidence (I² = 66.73%; GRADE: very low). When the analysis was restricted to studies with a low or moderate risk of bias (9 groups, 629 participants), the proportion was 4.78% (95% CI: 1.97‒11.12), with the quality of evidence remaining very low (I^2^ = 72.57%; GRADE: very low). No subgroup analysis for bradycardia, including specific definitions like a Heart Rate (HR) decrease of 20% from baseline, achieved a moderate or high quality of evidence. A multivariable meta-regression analysis did not find a significant correlation between the proportion of bradycardia and mean age, procedural invasiveness, IN-DEX dose, or the risk of bias score.

When the analysis was restricted to non-Chinese clinical trials (7 groups, 347 participants), the proportion of bradycardia was 9.12% (95% CI: 6.34‒12.95). Notably, this finding was supported by a moderate quality of evidence and demonstrated no heterogeneity among studies (I² = 0%; GRADE: moderate). A further sensitivity analysis within this non-Chinese subset, which excluded studies with a high risk of bias (4 groups, 198 participants), yielded a similar proportion of 9.73% (95% CI: 6.21‒14.94). This more restricted analysis also showed no heterogeneity and was supported by a moderate quality of evidence (I^2^ = 0%; GRADE: moderate). Details are shown in Appendix A.

### Desaturation

The overall proportion of desaturation across 25 study groups (1,289 participants) was 2.76% (95% CI: 1.87‒4.06; 95% PI: 1.80‒5.19), a finding supported by moderate quality of evidence with no heterogeneity among studies (I^2^ = 0%; GRADE: moderate). This result remained robust across multiple sensitivity analyses. When restricted to studies with a low or moderate risk of bias, the proportion was 3.07% (95% CI: 1.90‒4.92), with the evidence of quality remaining moderate (I^2^ = 0%; GRADE: moderate). Similarly, in an analysis of non-Chinese trials, the proportion was 3.92% (95% CI: 2.51‒6.07), also with moderate quality of evidence and no heterogeneity (I^2^ = 0%; GRADE: moderate). A multivariable meta-regression did not find any significant correlation between the proportion of desaturation and mean age, procedural invasiveness, dexmedetomidine dose, or the risk of bias score.

### Risk of bias assessment

The methodological quality of the included studies was assessed using the RoB2 tool for randomized controlled trials. Overall, 9 studies (32.1%) were classified as having a low risk of bias, 4 (14.3%) raised some concerns, and 15 (53.6%) were deemed to have a high risk of bias. The domain concerning bias arising from the randomization process showed the lowest risk, with all 28 studies (100%) assessed as low risk. In contrast, the highest risks were identified in the domains of bias in selection of the reported result, where 16 studies (57.1%) were rated as high risk, and bias due to missing outcome data, where 12 studies (42.9%) were rated as high risk. A significant number of studies, 7 (25%), were also classified as high risk for bias due to deviations from intended interventions.

## Discussion

This meta-analysis yields three principal findings that clarify the clinical effects of Intranasal Dexmedetomidine (IN-DEX) in the pediatric population. First, the overall success rate, defined as a single application without the need for adjuvant sedatives, was approximately 80%. Notably, a dose of [2, 3) mcg.kg^-1^ was associated with an 84% success rate, supported by high-quality evidence. Second, the temporal profile of sedation was characterized by a mean onset time of approximately 20 minutes and a mean duration of about 60 minutes, although both outcomes exhibited substantial heterogeneity. Third, IN-DEX demonstrated a favorable respiratory profile with a low and consistent incidence of desaturation (∼3%). The most common adverse events were hemodynamic, including hypotension (∼8%) and bradycardia (∼5%), with hypotension being more frequent in younger patients.

The pooled overall success rate of approximately 80% identified in this meta-analysis is broadly consistent with the existing literature, yet it also highlights the significant variability in efficacy reported by individual observational studies, with rates ranging from 57% to 100%. A primary driver of this heterogeneity appears to be the diverse definitions of 'successful sedation' and the wide array of procedures performed. For instance, studies on minimally stimulating procedures like echocardiography or EEG reported success rates exceeding 95%, whereas studies involving longer and more stimulating procedures like MRI reported much lower efficacy for IN-DEX as a sole agent.^12^, ^13^, ^14^, ^15^ The criteria for success varied substantially, from achieving a specific score on a sedation scale (e.g., MOAA/S ≤ 3 or Ramsay ≥ 3), as seen in the studies by Li et al. and Saudek et al., to a more pragmatic endpoint of completing the entire procedure without the need for rescue sedatives, a common definition in studies on EEG and ABR.^16^, ^17^, ^18^, ^19^ Therefore, the pooled estimate of 80% provides a more clinically representative benchmark, averaging the effects across different procedural contexts and outcome definitions, and underscores the reliable efficacy of IN-DEX for a general pediatric population undergoing non-painful procedures.

While acknowledging the significant heterogeneity for temporal outcomes, this meta-analysis establishes a clinically relevant benchmark profile for IN-DEX, with a mean sedation onset of approximately 20 minutes and a duration of about 60 minutes. This variability in onset is complex; the meta-regression indicated that onset is prolonged by procedural invasiveness and likely higher patient anxiety, given that a heightened state of sympathetic arousal directly counteracts the central sympatholytic effect of dexmedetomidine. Furthermore, the analysis revealed that onset may be paradoxically delayed by higher doses, a finding attributable to several factors, including a potential dose-dependent vasoconstrictor effect or unmeasured confounders like varying drug concentrations.^20^ Despite this high variability, the pooled mean onset of approximately 20 minutes positions IN-DEX as significantly faster than older oral agents like triclofos sodium and generally faster than, or at least comparable to, oral chloral hydrate.^19^^,^^21^^,^^22^ This onset is predictably slower than that of other intranasal agents such as midazolam or ketamine, which typically take effect in under 15 minutes.^23^, ^24^, ^25^

Conversely, the key advantage of IN-DEX lies in its more sustained duration of action. While intranasal midazolam's effect is often brief, sometimes lasting less than 20 minutes, the pooled estimate of a ∼60-minute duration highlights IN-DEX's suitability for procedures that exceed a very short timeframe.^26^ This profile represents a favorable clinical trade-off: in exchange for a slightly longer waiting period for onset compared to some alternatives, clinicians achieve a more stable and prolonged plane of sedation, potentially reducing the need for redosing. An important nuance to this finding, however, comes from our meta-regression, which revealed that duration significantly increases with patient age. This is likely a pharmacokinetic phenomenon tied to body composition: as children age, their proportion of adipose tissue increases, enlarging the volume of distribution for lipophilic dexmedetomidine and prolonging its clinical effect.^27^ Furthermore, while combining IN-DEX with agents like ketamine or midazolam can shorten onset, this often comes at the cost of significantly prolonged recovery and discharge times, reinforcing the efficiency of IN-DEX monotherapy for procedures of moderate length.^18^^,^^28^

Regarding safety, the findings from this meta-analysis reinforce the characteristic profile of IN-DEX, which is marked by a notable dissociation between its respiratory and hemodynamic effects. The low pooled incidence of oxygen desaturation (∼3%) is a key finding, underscoring its reputation for respiratory stability. This is consistent with data from numerous comparative trials where IN-DEX demonstrated a similar or often superior respiratory safety profile compared to agents like oral chloral hydrate, midazolam, and ketamine.^29^, ^30^, ^31^ This respiratory-sparing effect is a direct consequence of its α2-adrenergic agonist mechanism, which differs fundamentally from GABAergic or opioid agents.

In contrast, the most frequently observed adverse events were hemodynamic, with pooled incidences of approximately 8% for hypotension and 5% for bradycardia. Given the low certainty of the evidence, these numbers should be viewed with caution, as estimates that may change with more rigorous future research. Beyond the issue of generalizability, the significant geographic concentration of studies raises potential pharmacogenomic concerns. Dexmedetomidine is primarily metabolized by hepatic cytochrome P450 enzymes, particularly CYP2A6, which is known to have genetic polymorphisms that vary in prevalence across different ethnic populations.^32^ These genetic variations can influence drug clearance, potentially affecting the incidence of adverse events. Therefore, the safety profile identified in this meta-analysis may not be directly applicable to all pediatric populations. While these effects are pharmacologically expected, it is crucial to note that the vast majority of these events reported in the literature were transient, mild, and self-resolving, rarely requiring clinical intervention.^3^^,^^21^^,^^26^^,^^30^^,^^33^ Furthermore, IN-DEX was associated with a lower incidence of other troublesome side effects, particularly vomiting, when compared to traditional agents like chloral hydrate.^29^^,^^34^ This overall safety profile suggests that while vigilant hemodynamic monitoring is essential during IN-DEX sedation, its advantages in respiratory stability and reduced gastrointestinal side effects make it a highly favorable option for pediatric procedural sedation.

## Limitations

This meta-analysis has several important limitations. First, substantial heterogeneity was observed for temporal outcomes like sedation onset and duration (I^2^ > 85%), likely stemming from diverse administration techniques and patient populations; therefore, these pooled estimates should be interpreted as an average value. Second, the evidence base is constrained by a significant geographic concentration in Asia, primarily China, and a high proportion of studies with a high risk of bias (57.4%). While our sensitivity analyses confirmed the robustness of the primary efficacy outcomes after excluding these respective study groups, the geographic bias may limit the generalizability of safety findings, and the overall poor quality of the primary evidence warrants a cautious interpretation. Finally, the statistical power to draw firm conclusions for certain subgroups was limited, particularly for lower doses in the [1, 2) mcg.kg^-1^ range and for patients undergoing more invasive procedures, meaning these specific findings must be considered exploratory.

## Conclusion

In conclusion, this meta-analysis provides high-quality evidence that IN-DEX, at doses of [2, 3) mcg.kg^-1^, is highly effective for non-painful procedural sedation in the pediatric population. Although the evidence for its temporal profile is of low quality, the pooled estimates for sedation onset and duration still serve as a useful framework for clinical planning. However, every case must be individualized. The use of IN-DEX carries a non-negotiable requirement for adequate hemodynamic monitoring, including regular assessment of blood pressure and heart rate. Ultimately, clinicians must be prepared to manage its potential adverse effects to ensure patient safety.

## Data availability statement

The datasets generated and/or analyzed during the current study are available from the corresponding author upon reasonable request.

## Study registration

This study is a systematic review and meta-analysis of published data and, therefore, does not require institutional review board approval for its conduct. It was prospectively registered in the International Prospective Register of Systematic Reviews (PROSPERO) under the ID CRD420250652456

## AI assistance disclosure

No AI tools were used in the preparation or analysis of this manuscript. The authors take full responsibility for the content.

## Authors’ contributions

Kelvin Oliveira Rocha: Study conception, search strategy, initial screening, risk of bias assessment, statistical analysis, data interpretation, manuscript writing, and revision.

Ellen Renata Ferreira de Araújo Santos: Resolution of screening discrepancies, critical revision of the manuscript.

Mariana Madureira Pombeiro: Initial screening, risk of bias assessment, critical revision of the manuscript.

Márcia Nayara da Silva Leite Fidelis: Critical revision of the manuscript and contribution to clinical expertise.

Fabiana Maria Kakehasi: Overall supervision, critical revision of the manuscript.

Daniela Caldas Teixeira: Overall supervision, critical revision of the manuscript.

## Funding

No funding was received for conducting this study.

## Conflicts of interest

The authors declare no conflicts of interest.

## Appendix Supplementary materials

Image, application 1

## Footnotes

Supplementary material associated with this article can be found in the online version at doi:10.1016/j.bjane.2025.844717.
