# Supplementary material for: Intranasal dexmedetomidine for procedural sedation in children: a systematic review and meta-analysis
Source: Braz J Anesthesiol. 2025 Dec 5;76(1):844717. doi: 10.1016/j.bjane.2025.844717 (PMC12797056; doi:10.1016/j.bjane.2025.844717)
Supplement: Supplementary file 1 [file mmc1.docx]

**BJAN-D-25-00447_Supplementary Material**

**APPENDIX A**

Contents

[Search Strategy 3](#_Toc213970135)

[Clinical trials assessment 3](#_Toc213970136)

[Data Distribution 3](#_Toc213970137)

[Overview of Definitions 6](#_Toc213970138)

[Hypotension 6](#_Toc213970139)

[Bradycardia 6](#_Toc213970140)

[Dessaturation 6](#_Toc213970141)

[Onset Time / Duration 7](#_Toc213970142)

[Onset Time 7](#_Toc213970143)

[Sensitivity Analysis of Onset Time Restricted to Studies Without High Risk of Bias According to RoB2 7](#_Toc213970144)

[Meta-Regression of Onset Time (Restricted to Studies with Low or Moderate Risk of Bias) 7](#_Toc213970145)

[Analysis of Onset Time Restricted to Non-Chinese Clinical Trials 8](#_Toc213970146)

[Duration Time 8](#_Toc213970147)

[Sensitivity Analysis of Duration Time Restricted to Studies Without High Risk of Bias According to RoB2 8](#_Toc213970148)

[Meta-Regression of Duration Time (Restricted to Studies with Low or Moderate Risk of Bias) 9](#_Toc213970149)

[Analysis of Duration Time Restricted to Non-Chinese Clinical Trials 9](#_Toc213970150)

[Success Rates 10](#_Toc213970151)

[Evaluation of All Included Clinical Trial Studies 10](#_Toc213970152)

[Subgroup Assessment Based on the Full Set of Clinical Trials 10](#_Toc213970153)

[Metarregression Success vs Mean Age + Invasiveness Level + DEX dose + RoB2 score 10](#_Toc213970154)

[Subgroup Analysis Excluding Studies with High Risk of Bias 11](#_Toc213970155)

[Key Adverse Events: Hypotension, Bradycardia, and Desaturation 13](#_Toc213970156)

[Evaluation of All Included Clinical Trial Studies 13](#_Toc213970157)

[Hypotension 13](#_Toc213970158)

[Subgroup Assessment Based on the Full Set of Clinical Trials 13](#_Toc213970159)

[Metarregression Hypotension vs Mean Age + Invasiveness Level + DEX dose + RoB2 score 13](#_Toc213970160)

[Subgroup Analysis Excluding Studies with High Risk of Bias 14](#_Toc213970161)

[Bradycardia 14](#_Toc213970162)

[Subgroup Assessment Based on the Full Set of Clinical Trials 14](#_Toc213970163)

[Metarregression Bradycardia vs Mean Age + Invasiveness Level + DEX dose + RoB2 score 15](#_Toc213970164)

[Dessaturation 15](#_Toc213970165)

[Subgroup Assessment Based on the Full Set of Clinical Trials 15](#_Toc213970166)

[Metarregression Dessaturation vs Mean Age + Invasiveness Level + DEX dose + RoB2 score 16](#_Toc213970167)

[Subgroup Analysis Excluding Studies with High Risk of Bias 17](#_Toc213970168)

[Evaluation Restricted to Non-Chinese Studies 17](#_Toc213970169)

[Hypotension 18](#_Toc213970170)

[Subgroup Assessment Based on the non-Chinese Clinical Trials 18](#_Toc213970171)

[Metarregression Hypotension vs Mean Age + Invasiveness Level + DEX dose + RoB2 score 19](#_Toc213970172)

[Bradycardia 19](#_Toc213970173)

[Subgroup Assessment Based on the non-Chinese Clinical Trials 19](#_Toc213970174)

[Metarregression Bradycardia vs Mean Age + Invasiveness Level + DEX dose + RoB2 score 20](#_Toc213970175)

[Dessaturation 20](#_Toc213970176)

[Subgroup Assessment Based on the non-Chinese Clinical Trials 20](#_Toc213970177)

[Metarregression Dessaturation vs Mean Age + Invasiveness Level + DEX dose + RoB2 score 21](#_Toc213970178)

[Risk of Bias Assessment 22](#_Toc213970179)

[GRADE presentation 22](#_Toc213970180)

**1. Search Strategy**

**Pubmed:** (pediatrics OR children OR infants OR adolescents OR neonates OR toddlers) AND (dexmedetomidine OR "alpha-2 adrenergic agonist") AND (Nasal OR Intranasal).

**ScienceDirect:** (pediatrics OR infants OR neonatal) AND (dexmedetomidine OR "Precedex") AND (Nasal OR Intranasal).

**Scielo:** (pediatrics OR infants OR neonatal) AND (dexmedetomidine OR "Precedex") AND (Nasal OR Intranasal)

**2. Clinical trials assessment**

2.1. Data Distribution

**Figure** Distribution of the number of studies and participant counts by country, stratified by study type.

**Figure** Participant distribution according to pediatric age strata, defined by the mean age reported in each study.


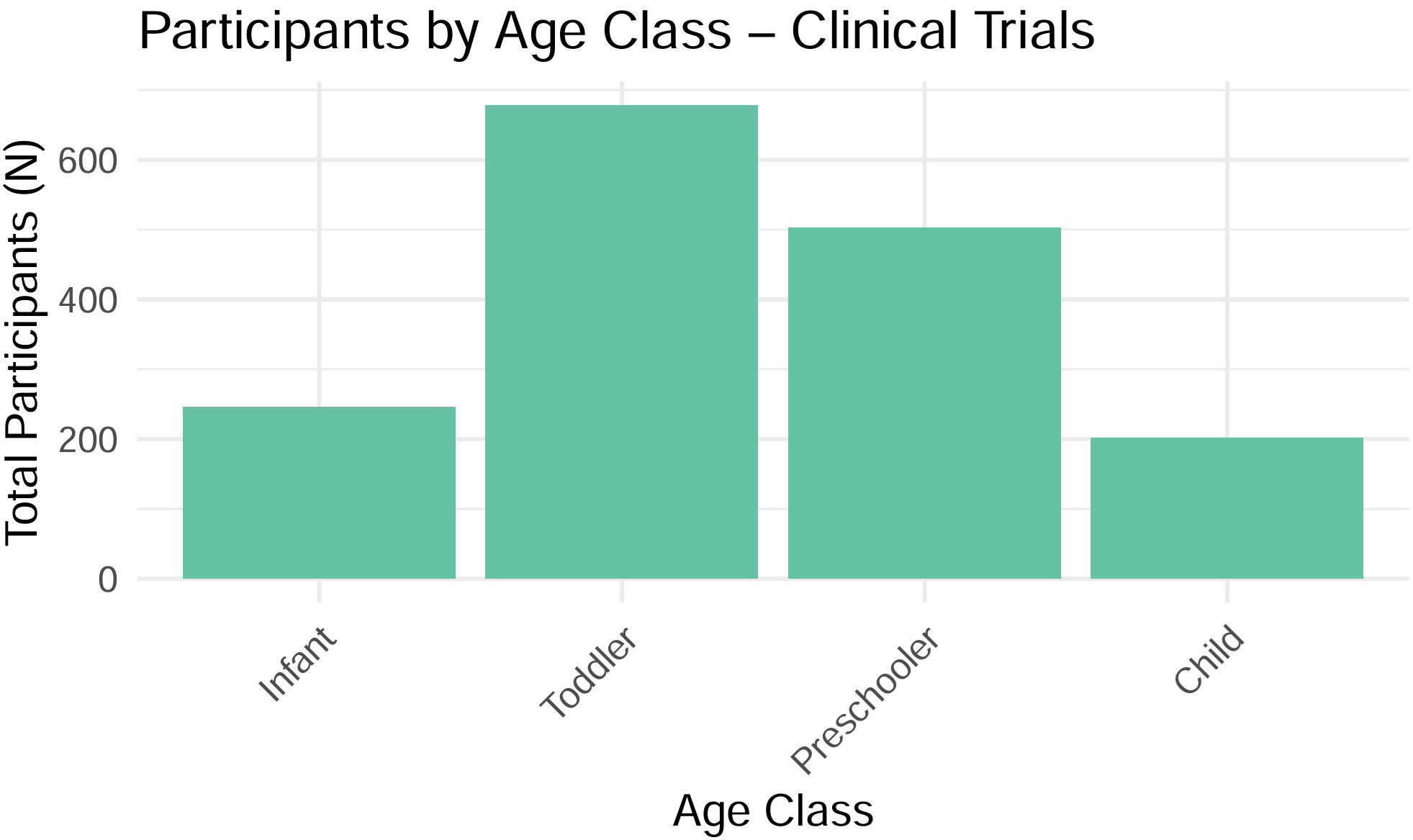


**Figure** Distribution of participant numbers across invasiveness level strata. Procedures were classified into four levels of invasiveness based on degree of physical contact, tissue penetration, and pain potential, as defined in the main manuscript.


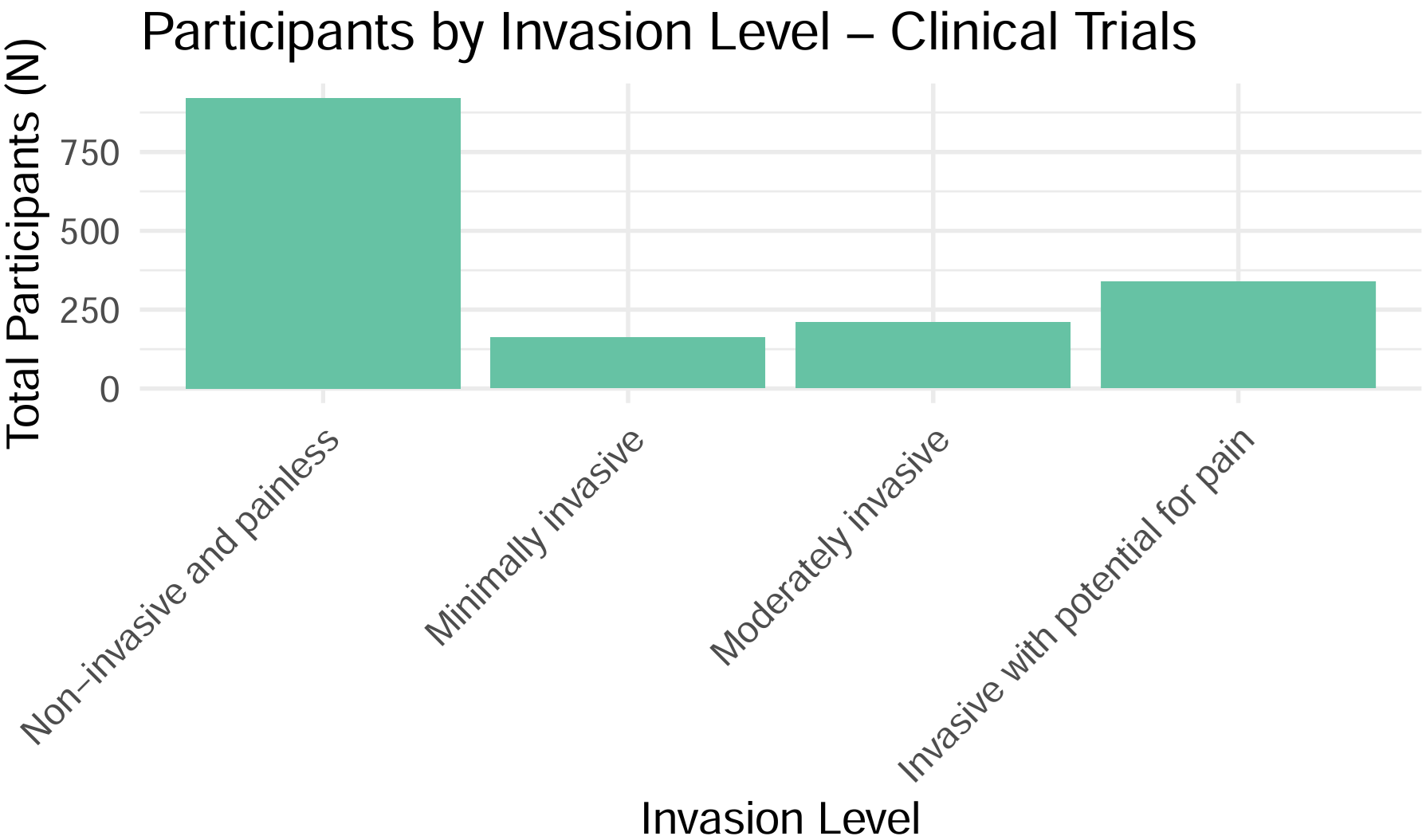


**Figure** Participant distribution by intranasal dexmedetomidine dose ranges.


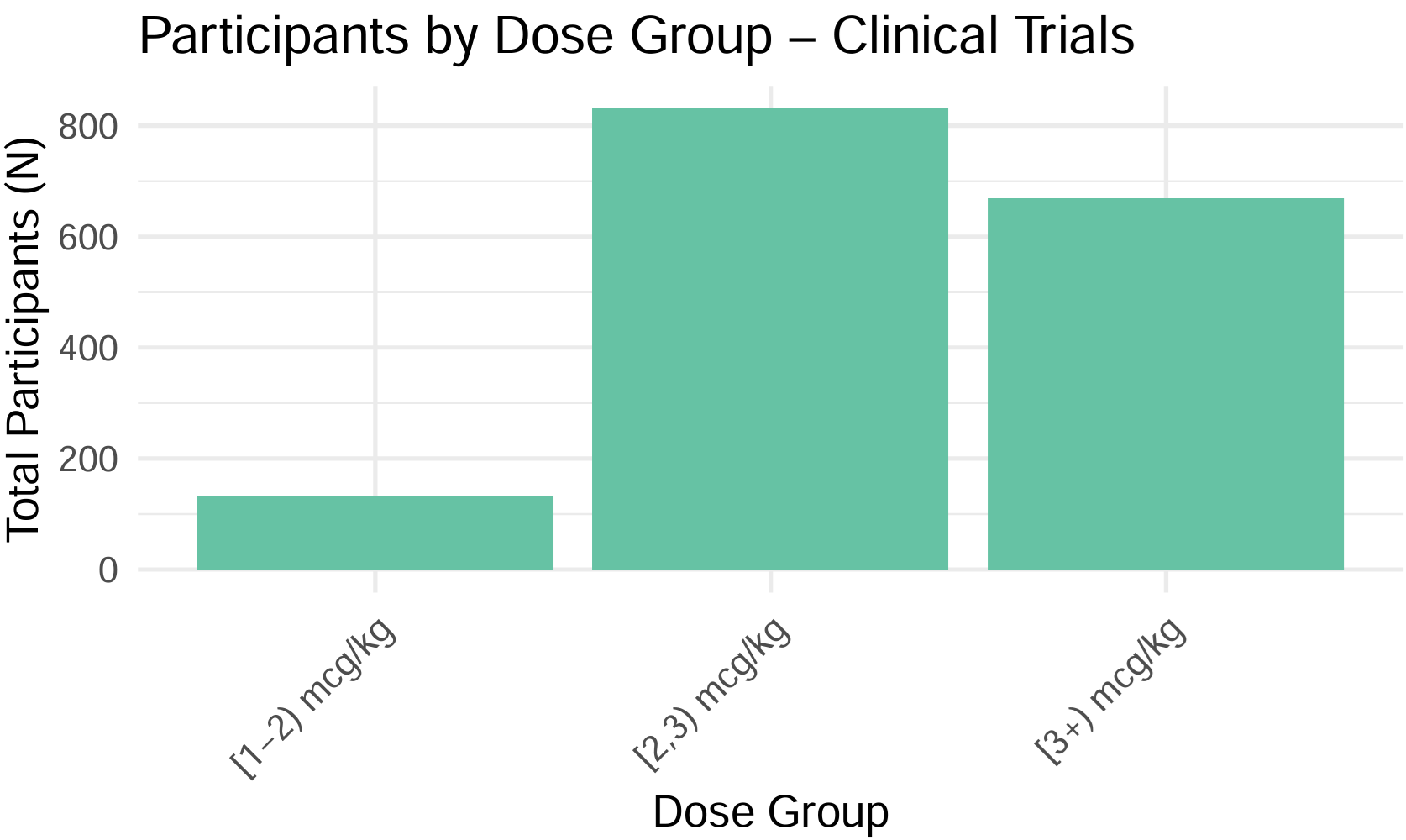


2.1.1. Overview of definitions

Hypotension

**Table** Summary of hypotension definitions across included studies.

|  | **Clinical trial** | **Observational** |
| --- | --- | --- |
|  | **Distinct Groups (n)** | **Distinct Groups (n)** |
| **NA** | 26 (1032) | 7 (4144) |
| **SBP < 20% basal** | 8 (479) | 5 (189) |
| **SBP < 70** | 1 (118) | 3 (85) |
| **MSBP < SD idade** | ‒ | 2 (200) |
| **SBP < 30% basal** | ‒ | 1 (371) |

SBP, Systolic Blood Pressure; MSBP, Mean Systolic Blood Pressure; NA, Not Applicable.

Bradycardia

**Table** Summary of bradycardia definitions across included studies.

|  | **Clinical trial** | **Observational** |
| --- | --- | --- |
|  | **Distinct Groups (n)** | **Distinct Groups (n)** |
| **NA** | 22 (790) | 1 (371) |
| **HB < 20% basal** | 7 (535) | 7 (4037) |
| **HB < 60 bpm** | 4 (136) | 5 (278) |
| **HB < 80 bpm** | 2 (168) | 2 (25) |
| **HB < 2 SD to age** | ‒ | 2 (200) |
| **HB < 100 bpm** | ‒ | 1 (78) |

HC, Heartbeat; SD, Standard-Deviation; NA, Not Applicable.

Dessaturation

**Table** Summary of bradycardia definitions across included studies.

|  | **Clinical trial** | **Observational** |
| --- | --- | --- |
|  | **Distinct Groups (n)** | **Distinct Groups (n)** |
| **NA** | 10 (340) | 2 (83) |
| **SpO_2_ < 94%** | 12 (562) | 1 (60) |
| **SpO_2_ < 92%** | 7 (536) | 4 (166) |
| **SpO_2_ < 90%** | 5 (147) | 11 (4680) |
| **SpO_2_ < 88%** | 1 (44) | - |

SpO_2_, Peripheral Oxygen Saturation; NA, Not Applicable.

2.2. Onset time / Duration

2.2.1. Onset time

Sensitivity analysis of onset time restricted to studies without high-risk of bias according to RoB2

**Table** Sensitivity assessment of onset time across clinical trial publications – Restricted to studies without high-risk of bias according to RoB2.

| **Variables** | **K (Events / N)** | **Mean (95% CI)** | **I^2^** | **Ajusted Mean (95% CI)** | **GRADE** |
| --- | --- | --- | --- | --- | --- |
| Onset Time General (high risk included) | 34 (34/1609) | 18.9 (16.6 ‒ 21.4) | 99.3 | 15.5 (13.6 ‒ 17.8) | Very low |
| High risk of bias excluded | 16 (16/897) | 20.5 (17.3 ‒ 24.3) | 98.6 | 22.4 (19.0 ‒ 26.4) | Low |
| Infant | 2 (2/146) | 16.9 (12.3 ‒ 23.2) | 97.4 | ‒ | Very low |
| Toddler | 7 (7/489) | 17.4 (15.2 ‒ 20.0) | 95.3 | 17.4 (15.2 ‒ 20.0) | Low |
| Preschooler | 6 (6/217) | 28.0 (20.8 ‒ 37.5) | 98.6 | 29.2 (22.3 ‒ 38.3) | Very low |
| Non-invasive and painless | 12 (12/733) | 19.2 (17.1 ‒ 21.5) | 95.3 | 18.6 (16.5 ‒ 20.8) | Low |
| Moderately invasive | 2 (2/106) | 23.0 (21.4 ‒ 24.7) | 67.1 | **‒** | Low |
| Invasive with potential for pain | 2 (2/58) | 25.7 (8.8 ‒ 74.7) | 99.7 | ‒ | Very low |
| Dose: [2, 3) mcg.kg^-1^ | 9 (9/442) | 19.7 (15.1 ‒ 25.7) | 99.1 | 24.2 (18.8 ‒ 31.2) | Very low |
| Dose: ≥ 3 mcg.kg^-1^ | 7 (7/455) | 21.4 (18.2 ‒ 25.0) | 95.7 | 18.8 (15.9 ‒ 22.2) | Low |

General, Overall group encompassing all event subgroups definitions; K, Distinct Subgroups; SBP, Systolic Blood Pressure; HR, Heart Rate.

Adjusted proportions represent the estimated values following correction using the trim and fill method in groups showing evidence of publication bias.

Meta-regression of onset time (restricted to studies with low or moderate risk of Bias)

**Table** Meta-regression analysis of sedation onset time, restricted to studies not classified as high-risk by the RoB2 tool.

| **Variável** | **ExpB** | **B (SE)** | **p-val** | **I^2^** | **R^2^** |
| --- | --- | --- | --- | --- | --- |
| Intercept | 6.54 | 1.878 (0.365) | < 0.001 | 97.48714 | 46.44577 |
| Mean Age | 1.05 | 0.052 (0.035) | 0.139 |  |  |
| Invasiveness level | 1.18 | 0.166 (0.064) | **0.009** |  |  |
| DEX dose | 1.4 | 0.340 (0.131) | **0.01** |  |  |

The meta-regression indicates that sedation onset time increases with both procedural invasiveness and intranasal dexmedetomidine dosage.

Analysis of onset time restricted to non-Chinese clinical trials

**Table** Sensitivity assessment of onset time restricted to non-Chinese clinical trials.

| **Variáveis** | **K (Events / N)** | **Mean (95% CI)** | **I^2^** | **Ajusted Mean (95% CI)** | **GRADE** |
| --- | --- | --- | --- | --- | --- |
| Onset Time General (high risk included) | 18 (18/687) | 18.9 (15.0 ‒ 23.8) | 99.5 | 14.3 (11.2 ‒ 18.1) | Very low |
| High risk of bias excluded | 7 (7/318) | 24.7 (17.4 ‒ 35.0) | 99.0 | 34.3 (23.0 ‒ 50.9) | Very low |
| Toddler | 2 (2/89) | 17.9 (9.6 ‒ 33.3) | 98.8 | NA | Very low |
| Preschooler | 4 (4/111) | 31.0 (22.3 ‒ 43.2) | 95.6 | 41.5 (30.5 ‒ 56.5) | Very low |
| Non-invasive and painless | 6 (6/274) | 22.2 (17.8 ‒ 27.8) | 96.2 | 20.9 (16.9 ‒ 25.8) | Low |
| Dose: [2,3) mcg.kg^-1^ | 4 (4/249) | 22.3 (12.7 ‒ 39.2) | 99.5 | 27.5 (16.1 ‒ 46.7) | Very low |
| Dose: ≥ 3 mcg.kg^-1^ | 3 (3/69) | 27.7 (23.5 ‒ 32.7) | 75.3 | 24.5 (20.8 ‒ 29.0) | Moderate |

General, Overall group encompassing all event subgroups definitions; K, Distinct subgroups; SBP, Systolic Blood Pressure; HR, heart rate.

Adjusted proportions represent the estimated values following correction using the trim and fill method in groups showing evidence of publication bias.

2.2.2. Duration time

Sensitivity analysis of duration time restricted to studies without high risk of bias according to RoB2

**Table** Sensitivity assessment of duration time across clinical trial publications – Restricted to studies without high-risk of bias according to RoB2.

| **Variáveis** | **K (Events / N)** | **Mean (95% CI)** | **I^2^** | **Ajusted Mean (95% CI)** | **GRADE** |
| --- | --- | --- | --- | --- | --- |
| Duration time general (high-risk included) | 28 (28/1368) | 60.3 (52.7 ‒ 69.1) | 99.3 | 60.3 (52.7 ‒ 69.1) | Low |
| High-risk of bias excluded | 11 (11/674) | 54.6 (47.8 ‒ 62.4) | 97.2 | 49.0 (42.6 ‒ 56.2) | Low |
| Infant | 2 (2/146) | 46.0 (34.7 ‒ 60.9) | 95.6 | ‒ | Very low |
| Toddler | 4 (4/372) | 51.0 (40.9 ‒ 63.6) | 97.3 | 44.8 (34.6 ‒ 58.0) | Very low |
| Preschooler | 4 (4/111) | 61.9 (42.8 ‒ 89.7) | 97.5 | 49.3 (33.7 ‒ 72.1) | Very low |
| Non-invasive and painless | 10 (10/630) | 56.5 (49.1 ‒ 64.9) | 97.2 | 50.0 (43.4 ‒ 57.7) | Very low |
| Dose: [2,3) mcg.kg^-1^ | 6 (6/322) | 54.0 (44.5 ‒ 65.5) | 96.9 | 54.0 (44.5 ‒ 65.5) | Low |
| Dose: ≥ 3 mcg.kg^-1^ | 5 (5/352) | 55.5 (44.6 ‒ 69.1) | 97.4 | 45.9 (36.6 ‒ 57.5) | Very low |

All variables following “High-risk of bias excluded” refer specifically to studies classified as having low or moderate risk of bias.

General, Overall group encompassing all event subgroups definitions; K, Distinct subgroups; SBP, Systolic Blood Pressure; HR, Heart Rate.

Adjusted proportions represent the estimated values following correction using the trim and fill method in groups showing evidence of publication bias.

Meta-regression of duration time (restricted to studies with low or moderate risk of Bias)

**Table** Meta-regression analysis of sedation duration time, restricted to studies not classified as high-risk by the RoB2 tool.

| **Variável** | **ExpB** | **B (SE)** | **p-val** | **I^2^** | **R^2^** |
| --- | --- | --- | --- | --- | --- |
| Intercept | 51.49 | 3.941 (0.280) | < 0.001 | 95.5 | 37.2 |
| Mean Age | 1.06 | 0.062 (0.026) | **0.018** |  |  |
| Invasiveness level | 0.9 | -0.101 (0.065) | 0.118 |  |  |
| DEX dose | 0.92 | -0.078 (0.103) | 0.446 |  |  |

The meta-regression indicates that sedation duration time increases with mean age.

Analysis of duration time restricted to non-Chinese clinical trials

**Table** Sensitivity assessment of duration time restricted to non-Chinese clinical trials.

| **Variáveis** | **K (Events / N)** | **Mean (95% CI)** | **I^2^** | **Ajusted Mean (95% CI)** | **GRADE** |
| --- | --- | --- | --- | --- | --- |
| Duration Time general (high risk included) | 16 (16/630) | 58.0 (50.4 ‒ 66.7) | 98.7 | 49.1 (42.3 ‒ 57.0) | Very low |
| High risk of bias excluded | 6 (6/279) | 59.2 (48.6 ‒ 72.2) | 96.3 | 52.4 (43.0 ‒ 63.9) | Very low |
| Preschooler | 4 (4/111) | 61.9 (42.8 ‒ 89.7) | 97.5 | 49.3 (33.7 ‒ 72.1) | Very low |
| Non-invasive and painless | 5 (5/235) | 64.5 (52.4 ‒ 79.3) | 95.4 | 53.4 (44.2 ‒ 64.5) | Very low |
| Dose: [2,3) mcg.kg^-1^ | 4 (4/249) | 55.6 (42.9 ‒ 72.1) | 97.5 | 50.0 (38.3 ‒ 65.3) | Very low |
| Dose: ≥ 3 mcg.kg^-1^ | 2 (2/30) | 66.0 (59.6 ‒ 73.2) | 0 | ‒ | Very low |

All variables following “High risk of bias excluded” refer specifically to studies classified as having low or moderate risk of bias.

General, Overall group encompassing all event subgroups definitions; K, Distinct subgroups; SBP, Systolic Blood Pressure; HR, Heart Rate.

Adjusted proportions represent the estimated values following correction using the *trim and fill* method in groups showing evidence of publication biasodo trim and fill.

2.3. Success rates

Procedural success was defined as the ability to complete the intervention without administering supplemental sedatives or repeating the initial sedation dose. The following results are restricted to studies meeting this criterion.

2.3.1. Evaluation of all included clinical trial studies

Subgroup assessment based on the full set of clinical trials

**Table** Sensitivity assessment of success rates across clinical trial publications.

| **Variables** | **K (Events / N)** | **Proportion** | **I^2^** | **Ajusted Proportion** | **GRADE** |
| --- | --- | --- | --- | --- | --- |
| Success general | 17 (897 / 1132) | 79.58% (73.56 ‒ 84.52) | 77.33 | 76.54% (70.21 ‒ 81.88) | Low |
| High risk of bias excluded | 12 (697 / 893) | 78.23% (70.31 ‒ 84.51) | 81.4 | 75.02% (66.81 ‒ 81.76) | Low |
| Infant | 2 (146 / 170) | 87.08% (75.16 ‒ 93.76) | 35.94 | ‒ | Low |
| Toddler | 7 (500 / 633) | 80.89% (73.04 ‒ 86.87) | 76.55 | 76.46% (68.19 ‒ 83.11) | Very low |
| Preschooler | 6 (181 / 244) | 72.52% (53.78 ‒ 85.68) | 85.15 | 72.52% (53.78 ‒ 85.68) | Very low |
| Child | 2 (70 / 85) | 82.17% (72.48 ‒ 88.96) | 0 | ‒ | Low |
| Non-invasive and painless | 12 (743 / 945) | 78.71% (71.02 ‒ 84.79) | 82.44 | 75.08% (67.05 ‒ 81.69) | Very low |
| Moderately invasive | 3 (79 / 101) | 77.92% (64.92 ‒ 87.07) | 42.49 | 66.67% (47.79 ‒ 81.38) | Very low |
| Dose: [2,3) mcg.kg^-1^ | 8 (357 / 429) | 82.45% (77.46 ‒ 86.52) | 25.12 | 81.25% (75.70 ‒ 85.77) | **Moderate** |

General, Overall group encompassing all event definitions; K, Distinct subgroups; SBP, Systolic Blood Pressure; HR, Heart Rate.

Adjusted proportions represent the estimated values following correction using the *trim and fill* method in groups showing evidence of publication bias.

Meta regression success vs. mean age + invasiveness level + DEX dose + RoB2 score

**Table** Multivariable meta-regression of success vs. mean age, invasiveness level, dexmedetomidine dose, and Risk of Bias Score (RoB2).

| **Variable** | **ExpB** | **B (SE)** | **p-val** | **I^2^** | **R^2^** |
| --- | --- | --- | --- | --- | --- |
| Intercept | 0.95 | 2.947 (1.077) | 0.006 | 78.2 | 0 |
| Mean Age | 0.477 | -0.092 (0.091) | 0.317 |  |  |
| Invasiveness level | 0.531 | 0.126 (0.279) | 0.653 |  |  |
| DEX dose | 0.386 | -0.464 (0.371) | 0.21 |  |  |
| RoB2 Score | 0.478 | -0.088 (0.268) | 0.742 |  |  |

This table shows no correlation between bradycardia and the analyzed variables.

**Figure 1** Forest plot of the weighted success proportion in the general pediatric population receiving intranasal dexmedetomidine at 2–3 mcg.kg^-1^/dose.


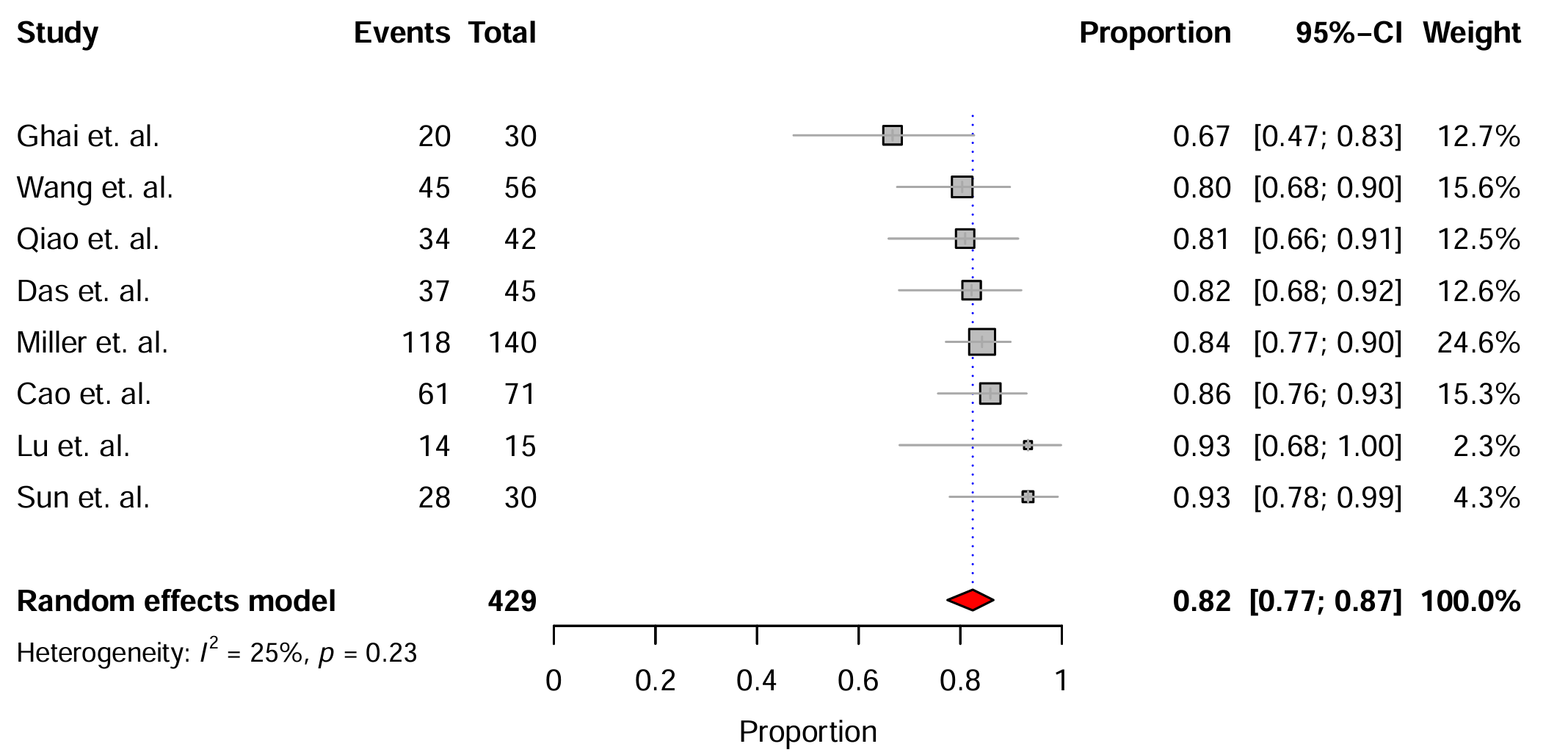


Subgroup analysis excluding studies with high risk of Bias

**Table** Sensitivity assessment of success rates across clinical trial publications – excluding studies high high risk of bias.

| **Variables** | **K (Events / N)** | **Proportion** | **I^2^** | **Ajusted Proportion** | **GRADE** |
| --- | --- | --- | --- | --- | --- |
| Success general | 12 (697 / 893) | 78.23% (70.31 ‒ 84.51) | 81.4 | 75.02% (66.81 ‒ 81.76) | Very low |
| Infant | 2 (146 / 170) | 87.08% (75.16 ‒ 93.76) | 35.94 | ‒ | Low |
| Toddler | 6 (439 / 562) | 79.96% (71.02 ‒ 86.66) | 77.97 | 76.64% (67.22 ‒ 83.99) | Very low |
| Preschooler | 3 (67 / 105) | 62.53% (29.44 ‒ 86.97) | 89.57 | 62.53% (29.44 ‒ 86.97) | Very low |
| Non-invasive and painless procedure | 11 (683 / 878) | 77.48% (69.29 ‒ 83.99) | 82.52 | 74.98% (66.58 ‒ 81.84) | Very low |
| Dose: [2,3) mcg.kg^-1^ | 5 (242 / 286) | 84.04% (79.21 ‒ 87.91) | 0 | 83.30% (77.95 ‒ 87.57) | **High** |
| Dose: ≥ 3 mcg.kg^-1^ | 7 (455 / 607) | 73.05% (60.78 ‒ 82.58) | 86.78 | 73.05% (60.78 ‒ 82.58) | Very low |

General, Overall group encompassing all event subgroups definitions; K, Distinct subgroups; SBP, Systolic Blood Pressure; HR, Heart Rate.

Adjusted proportions represent the estimated values following correction using the *trim and fill* method in groups showing evidence of publication bias.

**Figure 2** Forest plot of the weighted success proportion in pediatric patients receiving intranasal dexmedetomidine at [2–3) mcg.kg^-1^/dose, excluding high-risk studies according to RoB2.


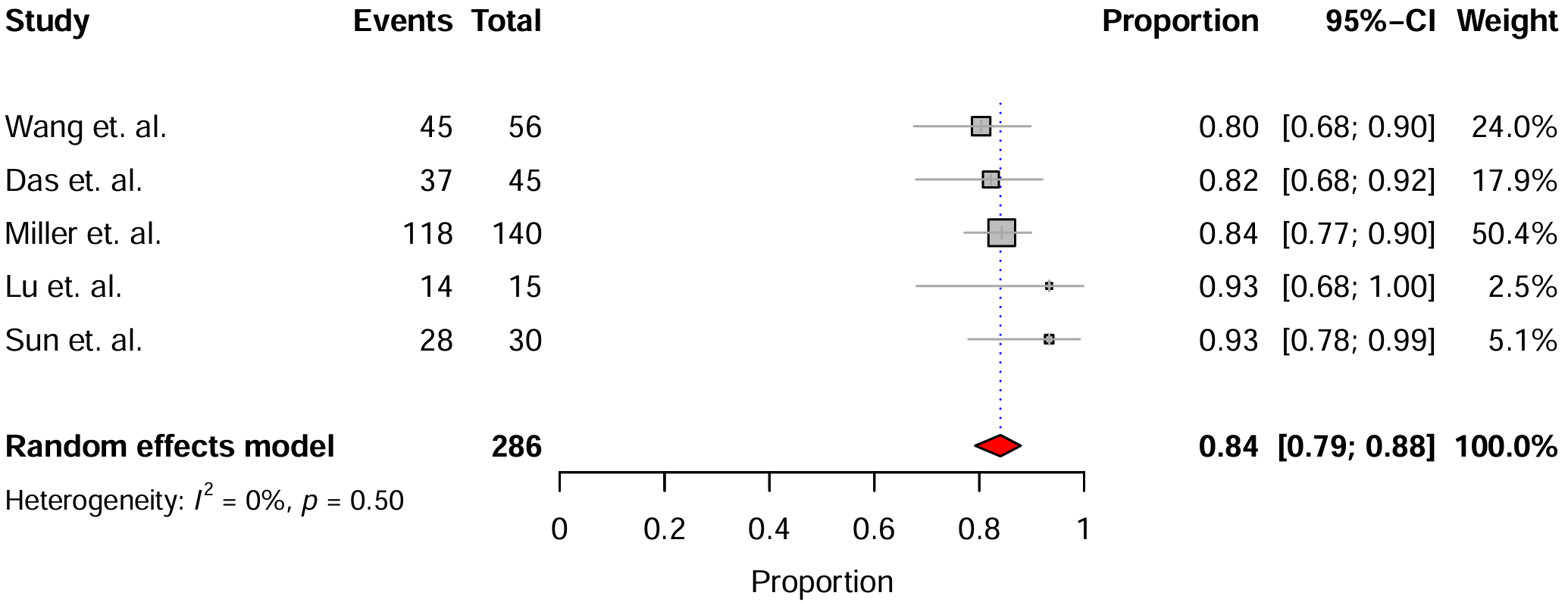


2.4. Key adverse events: hypotension, bradycardia, and desaturation

2.4.1 Evaluation of all included clinical trial studies

2.4.1.1. Hypotension

Subgroup assessment based on the full set of clinical trials

**Table** Sensitivity assessment of hypotension across clinical trial publications.

| **Variables** | **K (Events / N)** | **Proportion** | **I^2^** | **Ajusted Proportion** | **GRADE** |
| --- | --- | --- | --- | --- | --- |
| Hypotension general | 9 (52 / 597) | 8.24% (5.06 ‒ 13.16) | 55.81 | 10.19% (6.32 ‒ 16.02) | Very low |
| High risk of bias excluded | 5 (45 / 477) | 8.61% (4.59 ‒ 15.57) | 72.07 | 13.80% (7.79 ‒ 23.28) | Very low |
| Toddler | 3 (23 / 322) | 7.21% (4.83 ‒ 10.61) | 0 | 7.21% (4.83 ‒ 10.61) | Low |
| Preschooler | 2 (7 / 97) | 5.93% (0.76 ‒ 34.00) | 58.38 | ‒ | Very low |
| Non-invasive and painless procedures | 6 (52 / 537) | 9.30% (5.62 ‒ 15.01) | 65.14 | 13.20% (8.15 ‒ 20.68) | Very low |
| Dose: [2, 3) mcg.kg^-1^ | 2 (22 / 155) | 7.25% (0.52 ‒ 53.85) | 74.26 | ‒ | Very low |
| Dose: ≥ 3 mcg.kg^-1^ | 5 (30 / 407) | 7.85% (5.56 ‒ 10.99) | 0 | 8.66% (6.32 ‒ 11.75) | Very low |

General, Overall group encompassing all event definitions; K, Distinct subgroups; SBP, Systolic Blood Pressure; HR, Heart Rate.

Adjusted proportions represent the estimated values following correction using the *trim and fill* method in groups showing evidence of publication bias.

2.4.1.2. Meta regression hypotension vs. mean age + Invasiveness level + DEX dose + RoB2 score

**Table** Multivariable meta-regression of hypotension vs. mean age, invasiveness level, dexmedetomidine dose, and Risk of Bias Score (RoB2).

| **Variable** | **ExpB** | **B (SE)** | **p-val** | **I^2^** | **R^2^** |
| --- | --- | --- | --- | --- | --- |
| Intercept | 0.512 | 0.049 (1.712) | 0.977 | 0 | 100 |
| Mean Age | 0.302 | -0.836 (0.335) | **0.013** |  |  |
| Invasiveness level | 0.596 | 0.391 (0.772) | 0.613 |  |  |
| DEX dose | 0.434 | -0.265 (0.726) | 0.715 |  |  |
| RoB2 Score | 0.718 | 0.933 (0.405) | **0.021** |  |  |

This table provides evidence that both age and risk of bias score significantly influence the proportion of hypotension.

2.4.1.3. Subgroup analysis excluding studies with high-risk of bias

**Table** Sensitivity assessment of hypotension across clinical trial publications – excluding studies high high-risk of bias.

| **Variables** | **K (Events / N)** | **Proportion** | **I^2^** | **Ajusted Proportion** | **GRADE** |
| --- | --- | --- | --- | --- | --- |
| Hypotension general | 5 (45 / 477) | 8.61% (4.59 ‒ 15.57) | 72.07 | 13.80% (7.79 ‒ 23.28) | Very low |
| Toddler | 3 (23 / 322) | 7.21% (4.83 ‒ 10.61) | 0 | 7.21% (4.83 ‒ 10.61) | Low |
| Non-invasive and painless | 5 (45 / 477) | 8.61% (4.59 ‒ 15.57) | 72.07 | 13.80% (7.79 ‒ 23.28) | Very low |
| Dose: [2, 3) mcg.kg^-1^ | 2 (22 / 155) | 7.25% (0.52 ‒ 53.85) | 74.26 | ‒ | Very low |
| Dose: ≥ 3 mcg.kg^-1^ | 3 (23 / 322) | 7.21% (4.83 ‒ 10.61) | 0 | 7.21% (4.83 ‒ 10.61) | Low |

General, Overall group encompassing all event definitions; K, Distinct subgroups; SBP, Systolic Blood Pressure; HR, Heart Rate.

Adjusted proportions represent the estimated values following correction using the *trim and fill* method in groups showing evidence of publication bias.

**Table** Multivariable meta-regression of hypotension vs. mean age, invasiveness level, dexmedetomidine dose, and Risk of Bias Score (RoB2) – Excluding studies with high-risk of bias.

| **Variable** | **ExpB** | **B (SE)** | **p-val** | **I^2^** | **R^2^** |
| --- | --- | --- | --- | --- | --- |
| Intercept | 0.433 | 0.268 (1.977) | 0.892 | 0 | 100 |
| Mean Age | 0.298 | 0.857 (0.347) | **0.014** |  |  |
| Invasiveness level | 0.469 | 0.123 (0.836) | 0.883 |  |  |
| DEX dose | 0.586 | 0.349 (0.611) | 0.568 |  |  |
| RoB2 Score | 0.433 | 0.268 (1.977) | 0.892 |  |  |

This table provides evidence that age significantly influence the proportion of hypotension.

2.4.1.2. Bradycardia

Subgroup assessment based on the full set of clinical trials

**Table** Sensitivity assessment of bradycardia across clinical trial publications.

| **Variables** | **K (Events / N)** | **Proportion** | **I^2^** | **Ajusted Proportion** | **GRADE** |
| --- | --- | --- | --- | --- | --- |
| Bradycardia general | 13 (40 / 839) | 5.08% (2.61 ‒ 9.67) | 66.73 | 11.02% (5.62 ‒ 20.48) | Very low |
| High risk of bias excluded | 9 (31 / 629) | 4.78% (1.97 ‒ 11.12) | 72.57 | 11.71% (4.87 ‒ 25.55) | Very low |
| Toddler | 7 (21 / 547) | 2.67% (0.70 ‒ 9.61) | 80.09 | 14.12% (4.16 ‒ 38.39) | Very low |
| Child | 3 (9 / 144) | 7.71% (3.64 ‒ 15.62) | 14.07 | 9.46% (4.69 ‒ 18.17) | Very low |
| Non-invasive and painless procedures | 10 (31 / 679) | 4.20% (1.75 ‒ 9.76) | 72.16 | 11.89% (5.11 ‒ 25.28) | Very low |
| Dose: [2, 3) mcg.kg^-1^ | 6 (24 / 398) | 7.21% (3.78 ‒ 13.34) | 48.12 | 10.27% (4.94 ‒ 20.14) | Very low |
| Dose: ≥ 3 mcg.kg^-1^ | 7 (16 / 441) | 2.93% (0.68 ‒ 11.80) | 75.98 | 15.51% (4.16 ‒ 43.69) | Very low |

General, Overall group encompassing all event definitions; K, Distinct subgroups; SBP, Systolic Blood Pressure; HR, Heart Rate.

Adjusted proportions represent the estimated values following correction using the *trim and fill* method in groups showing evidence of publication bias.

Meta regression bradycardia vs. mean age + Invasiveness level + DEX dose + RoB2 score

**Table** Multivariable meta-regression of bradycardia vs. mean age, invasiveness level, dexmedetomidine dose, and Risk of Bias Score (RoB2).

| **Variable** | **ExpB** | **B (SE)** | **p-val** | **I^2^** | **R^2^** |
| --- | --- | --- | --- | --- | --- |
| Intercept | 0.09 | -2.318 (2.617) | 0.376 | 75.5 | 0 |
| Mean Age | 0.472 | -0.111 (0.271) | 0.681 |  |  |
| Invasiveness level | 0.715 | 0.921 (0.998) | 0.356 |  |  |
| DEX dose | 0.462 | -0.154 (0.887) | 0.862 |  |  |
| RoB2 Score | 0.343 | -0.648 (0.852) | 0.447 |  |  |

This table shows no correlation between bradycardia and the analyzed variables.

2.4.1.3. Dessaturation

Subgroup assessment based on the full set of clinical trials

**Table** Sensitivity assessment of dessaturation across clinical trial publications.

| **Variables** | **K (Events / N)** | **Proportion** | **I^2^** | **Ajusted Proportion** | **GRADE** |
| --- | --- | --- | --- | --- | --- |
| Dessaturation general | 25 (15 / 1289) | 2.76% (1.87 ‒ 4.06) | 0 | 4.06% (2.80 ‒ 5.86) | **Moderate** |
| High risk of bias excluded | 14 (11 / 846) | 3.07% (1.90 ‒ 4.92) | 0 | 4.02% (2.24 ‒ 7.14) | **Moderate** |
| Infant | 4 (7 / 246) | 3.80% (1.91 ‒ 7.43) | 0 | 4.44% (2.33 ‒ 8.30) | Low |
| Toddler | 8 (5 / 586) | 1.84% (0.76 ‒ 4.39) | 26.78 | 3.62% (1.45 ‒ 8.74) | Low |
| Child | 6 (3 / 193) | 3.06% (1.33 ‒ 6.88) | 0 | 3.77% (1.81 ‒ 7.68) | Low |
| Non-invasive and painless | 13 (11 / 806) | 3.03% (1.82 ‒ 5.02) | 3.38 | 4.31% (2.31 ‒ 7.89) | Low |
| Minimally invasive | 3 (1 / 161) | 1.33% (0.33 ‒ 5.17) | 0 | 2.00% (0.65 ‒ 5.99) | Low |
| Invasive with potential for pain | 5 (3 / 167) | 3.22% (1.34 ‒ 7.50) | 0 | 3.94% (1.84 ‒ 8.22) | Low |
| Dose: [2,3) mcg.kg^-1^ | 11 (12 / 626) | 3.36% (2.07 ‒ 5.43) | 0 | 4.27% (2.71 ‒ 6.68) | **Moderate** |
| Dose: ≥ 3 mcg.kg^-1^ | 10 (3 / 590) | 1.79% (0.85 ‒ 3.71) | 0 | ‒ | **Moderate** |

General, Overall group encompassing all event definitions; K, Distinct subgroups; SBP, Systolic Blood Pressure; HR, Heart Rate.

Adjusted proportions represent the estimated values following correction using the *trim and fill* method in groups showing evidence of publication bias.

Meta regression dessaturation vs. mean age + Invasiveness level + DEX dose + RoB2 score

**Table** Multivariable meta-regression of dessaturation vs. mean age, invasiveness level, dexmedetomidine dose, and Risk of Bias Score (RoB2).

| **Variable** | **ExpB** | **B (SE)** | **p-val** | **I^2^** | **R^2^** |
| --- | --- | --- | --- | --- | --- |
| Intercept | 0.066 | -2.655 (1.134) | 0.019 | 0 | 0 |
| Mean Age | 0.503 | 0.013 (0.120) | 0.913 |  |  |
| Invasiveness level | 0.455 | -0.181 (0.337) | 0.59 |  |  |
| DEX dose | 0.414 | -0.345 (0.430) | 0.422 |  |  |
| RoB2 Score | 0.505 | 0.019 (0.292) | 0.949 |  |  |

This table shows no correlation between dessaturation and the analyzed variables.

Subgroup analysis excluding studies with high risk of bias

**Table** Sensitivity assessment of dessaturation across clinical trial publications ‒ excluding studies high-risk of bias.

| **Variables** | **K (Events / N)** | **Proportion** | **I^2^** | **Ajusted Proportion** | **GRADE** |
| --- | --- | --- | --- | --- | --- |
| Dessaturation general | 14 (11 / 846) | 3.07% (1.90 ‒ 4.92) | 0 | 4.02% (2.24 ‒ 7.14) | Moderate |
| Infant | 2 (6 / 146) | 4.67% (2.18 ‒ 9.73) | 0 | ‒ | Low |
| Toddler | 6 (5 / 475) | 1.94% (0.66 ‒ 5.53) | 40.14 | 4.20% (1.38 ‒ 12.12) | **Moderate** |
| Non-invasive and painless procedures | 11 (11 / 696) | 3.24% (1.87 ‒ 5.55) | 6.25 | 4.26% (2.16 ‒ 8.20) | **Moderate** |
| Dose: [2, 3) mcg.kg^-1^ | 7 (9 / 391) | 3.80% (2.14 ‒ 6.67) | 0 | 4.60% (2.58 ‒ 8.09) | **Moderate** |
| Dose: ≥ 3 mcg.kg^-1^ | 7 (2 / 455) | 1.88% (0.78 ‒ 4.45) | 0 | 3.23% (1.20 ‒ 8.44) | **Moderate** |

General, Overall group encompassing all event definitions; K, Distinct subgroups; SBP, Systolic Blood Pressure; HR, Heart Rate.

Adjusted proportions represent the estimated values following correction using the *trim and fill* method in groups showing evidence of publication bias.

2.4.2. Evaluation restricted to non-Chinese studies

This section presents findings from non-Chinese studies, as well as the corresponding sensitivity analysis.

**Table** Sensitivity analysis of adverse event rates in non-Chinese clinical trial studies.

| **Variables** | **K (Events / N)** | **Proportion** | **I^2^** | **Ajusted Proportion** | **GRADE** |
| --- | --- | --- | --- | --- | --- |
| Hypotension General | 6 (29 / 275) | 8.41% (3.72 ‒ 17.93) | 53.12 | 13.80% (6.14 ‒ 28.16) | Very low |
| SBP < 20% basal | 5 (7 / 157) | 5.37% (1.99 ‒ 13.72) | 22.57 | ‒ | Low |
| Bradycardia General | 7 (26 / 347) | 9.12% (6.34 ‒ 12.95) | 0 | 9.95% (6.69 ‒ 14.55) | **Moderate** |
| HR < 20% do basal | 3 (9 / 149) | 7.43% (3.27 ‒ 16.02) | 21.57 | 9.46% (4.46 ‒ 18.94) | Low |
| Dessaturation General | 14 (14 / 563) | 3.92% (2.51 ‒ 6.07) | 0 | 4.77% (3.16 ‒ 7.12) | **Moderate** |
| SpO_2_ < 94% | 5 (3 / 142) | 3.57% (1.49 ‒ 8.28) | 0 | 4.06% (1.84 ‒ 8.72) | Low |
| SpO_2_ < 92% | 5 (9 / 303) | 4.30% (2.36 ‒ 7.71) | 0 | 5.03% (2.56 ‒ 9.64) | Low |
| SpO_2_ < 90% | 3 (2 / 74) | 4.14% (1.34 ‒ 12.06) | 0 | 5.13% (1.95 ‒ 12.81) | Low |

General, Overall group encompassing all event definitions; K, Distinct subgroups; SBP, Systolic Blood Pressure; HR, Heart Rate.

Adjusted proportions represent the estimated values following correction using the *trim and fill* method in groups showing evidence of publication bias.

**Table** Sensitivity analysis of adverse event rates in non-Chinese clinical trial studies ‒ excluding studies high-risk of bias.

| **Variables** | **K (Events / N)** | **Proportion** | **I^2^** | **Ajusted Proportion** | **GRADE** |
| --- | --- | --- | --- | --- | --- |
| Hypotension general | 5 (45 / 477) | 8.61% (4.59 ‒ 15.57) | 72.07 | 13.80% (7.79 ‒ 23.28) | Very low |
| SBP < 20% basal | 4 (23 / 359) | 6.94% (4.68 ‒ 10.20) | 0 | 7.21% (4.88 ‒ 10.52) | **Moderate** |
| Bradycardia general | 9 (31 / 629) | 4.78% (1.97 ‒ 11.12) | 72.57 | 11.71% (4.87 ‒ 25.55) | Very low |
| HR < 20% do basal | 4 (14 / 386) | 1.56% (0.09 ‒ 21.22) | 86.38 | 11.83% (1.14 ‒ 60.90) | Very low |
| Dessaturation general | 14 (11 / 846) | 3.07% (1.90 ‒ 4.92) | 0 | 4.02% (2.24 ‒ 7.14) | **Moderate** |
| SpO_2_ < 92% | 4 (9 / 401) | 2.98% (1.01 ‒ 8.45) | 50.18 | 3.89% (1.24 ‒ 11.59) | Low |
| SpO_2_ < 90% | 3 (2 / 112) | 3.28% (1.06 ‒ 9.72) | 0 | 5.13% (1.96 ‒ 12.77) | Low |

General, Overall group encompassing all event definitions; K, Distinct subgroups; SBP, Systolic Blood Pressure; HR, Heart Rate.

Adjusted proportions represent the estimated values following correction using the *trim and fill* method in groups showing evidence of publication bias.

2.4.2.1. Hypotension

Subgroup assessment based on the non-Chinese clinical trials

**Table** Sensitivity assessment of hypotension across clinical trial publications.

| **Variables** | **K (Events / N)** | **Proportion** | **I^2^** | **Ajusted Proportion** | **GRADE** |
| --- | --- | --- | --- | --- | --- |
| Hypotension general | 6 (29 / 275) | 8.41% (3.72 ‒ 17.93) | 53.12 | 13.80% (6.14 ‒ 28.16) | Very low |
| High risk of bias excluded | 2 (22 / 155) | 7.25% (0.52 ‒ 53.85) | 74.26 | ‒ | Very low |
| Preschooler | 2 (7 / 97) | 5.93% (0.76 ‒ 34.00) | 58.38 | ‒ | Very low |
| Non-invasive and painless | 3 (29 / 215) | 12.82% (5.99 ‒ 25.34) | 59.41 | 18.64% (9.54 ‒ 33.23) | Very low |
| Dose: [2, 3) mcg.kg^-1^ | 2 (22 / 155) | 7.25% (0.52 ‒ 53.85) | 74.26 | ‒ | Very low |
| Dose: ≥ 3 mcg.kg^-1^ | 2 (7 / 85) | 7.68% (1.65 ‒ 29.22) | 39.51 | ‒ | Low |

General, Overall group encompassing all event definitions; K, Distinct subgroups; SBP, Systolic Blood Pressure; HR, Heart Rate.

Adjusted proportions represent the estimated values following correction using the *trim and fill* method in groups showing evidence of publication bias.

Metarregression hypotension vs. Mean age + Invasiveness level + DEX dose + RoB2 score

**Table** Multivariable meta-regression of hypotension vs. Mean age, invasiveness level, dexmedetomidine dose, and Risk of Bias Score (RoB2).

| **Variable** | **ExpB** | **B (SE)** | **p-val** | **I^2^** | **R^2^** |
| --- | --- | --- | --- | --- | --- |
| Intercept | 0.449 | -0.204 (3.149) | 0.948 | 0 | 100 |
| Mean Age | 0.286 | -0.913 (0.455) | **0.045** |  |  |
| Invasiveness level | 0.619 | 0.483 (0.799) | 0.545 |  |  |
| DEX dose | 0.47 | -0.120 (1.203) | 0.92 |  |  |
| RoB2 Score | 0.75 | 1.098 (0.821) | 0.181 |  |  |

This table provides evidence that mean age significantly influences the proportion of hypotension.

2.4.2.2. Bradycardia

Subgroup assessment based on the non-Chinese clinical trials

**Table** Sensitivity assessment of bradycardia across clinical trial publications.

| **Variables** | **K (Events / N)** | **Proportion** | **I^2^** | **Ajusted Proportion** | **GRADE** |
| --- | --- | --- | --- | --- | --- |
| Bradycardia general | 7 (26 / 347) | 9.12% (6.34 ‒ 12.95) | 0 | 9.95% (6.69 ‒ 14.55) | **Moderate** |
| High risk of bias excluded | 4 (17 / 198) | 9.73% (6.21 ‒ 14.94) | 0 | 10.38% (6.73 ‒ 15.69) | **Moderate** |
| Toddler | 2 (7 / 100) | 5.29% (0.39 ‒ 44.57) | 72.12 | ‒ | Very low |
| Child | 2 (9 / 99) | 9.11% (4.81 ‒ 16.59) | 0 | ‒ | Low |
| Non-invasive and painless | 5 (17 / 248) | 8.38% (4.46 ‒ 15.20) | 25.46 | 10.10% (4.86 ‒ 19.80) | Low |
| Dose: [2, 3) mcg.kg^-1^ | 4 (24 / 292) | 9.50% (5.91 ‒ 14.93) | 26.1 | 10.27% (5.75 ‒ 17.68) | Moderate |
| Dose: ≥ 3 mcg.kg^-1^ | 3 (2 / 55) | 5.97% (1.93 ‒ 16.98) | 0 | 8.00% (3.07 ‒ 19.27) | Very low |

General, Overall group encompassing all event definitions; K, Distinct subgroups; SBP, Systolic Blood Pressure; HR, Heart Rate.

Adjusted proportions represent the estimated values following correction using the *trim and fill* method in groups showing evidence of publication bias.

Metarregression bradycardia vs. Mean age + Invasiveness level + DEX dose + RoB2 score

**Table** Multivariable meta-regression of bradycardia vs. mean age, invasiveness level, dexmedetomidine dose, and Risk of Bias Score (RoB2).

| **Variable** | **ExpB** | **B (SE)** | **p-val** | **I^2^** | **R^2^** |
| --- | --- | --- | --- | --- | --- |
| Intercept | 0.301 | -0.844 (2.342) | 0.718 | 47 | 0 |
| Mean Age | 0.508 | 0.033 (0.764) | 0.966 |  |  |
| Invasiveness level | 0.573 | 0.296 (2.002) | 0.883 |  |  |
| DEX dose | 0.374 | -0.514 (1.141) | 0.653 |  |  |
| RoB2 Score | 0.335 | -0.684 (0.850) | 0.421 |  |  |

This table shows no correlation between bradycardia and the analyzed variables.

2.4.2.3. Dessaturation

Subgroup assessment based on the non-Chinese clinical trials

**Table** Sensitivity assessment of dessaturation across clinical trial publications.

| **Variables** | **K (Events / N)** | **Proportion** | **I^2^** | **Ajusted Proportion** | **GRADE** |
| --- | --- | --- | --- | --- | --- |
| Dessaturation general | 14 (14 / 563) | 3.92% (2.51 ‒ 6.07) | 0 | 4.77% (3.16 ‒ 7.12) | Moderate |
| High risk of bias excluded | 6 (11 / 281) | 4.82% (2.79 ‒ 8.20) | 0 | 4.82% (2.79 ‒ 8.20) | Moderate |
| Toddler | 3 (5 / 139) | 4.79% (2.09 ‒ 10.62) | 0 | 4.79% (2.09 ‒ 10.62) | Low |
| Child | 5 (3 / 148) | 3.40% (1.42 ‒ 7.91) | 0 | 3.94% (1.84 ‒ 8.23) | Low |
| Non‒invasive and painless | 7 (11 / 347) | 4.47% (2.61 ‒ 7.56) | 0 | 4.79% (2.83 ‒ 8.00) | Low |
| Invasive with potential for pain | 5 (3 / 167) | 3.22% (1.34 ‒ 7.50) | 0 | 3.94% (1.84 ‒ 8.22) | Low |
| Dose: [2, 3) mcg.kg^-1^ | 5 (12 / 336) | 4.43% (2.59 ‒ 7.48) | 0 | 5.01% (2.99 ‒ 8.26) | Low |
| Dose: ≥ 3 mcg.kg^-1^ | 5 (2 / 154) | 3.23% (1.21 ‒ 8.32) | 0 | 4.45% (1.87 ‒ 10.20) | Very low |

General, Overall group encompassing all event definitions; K, Distinct subgroups; SBP, Systolic Blood Pressure; HR, Heart Rate.

Adjusted proportions represent the estimated values following correction using the *trim and fill* method in groups showing evidence of publication bias.

Metarregression dessaturation vs. Mean age + Invasiveness level + DEX dose + RoB2 score

**Table** Multivariable meta-regression of hypotension vs. mean age, invasiveness level, dexmedetomidine dose, and Risk of Bias Score (RoB2).

| **Variable** | **ExpB** | **B (SE)** | **p-val** | **I^2^** | **R^2^** |
| --- | --- | --- | --- | --- | --- |
| Intercept | 0.186 | -1.479 (1.482) | 0.318 | 0 | 0 |
| Mean Age | 0.581 | 0.328 (0.285) | 0.25 |  |  |
| Invasiveness level | 0.32 | ‒0.754 (0.631) | 0.232 |  |  |
| DEX dose | 0.34 | ‒0.662 (0.604) | 0.273 |  |  |
| RoB2 Score | 0.341 | ‒0.658 (0.517) | 0.203 |  |  |

This table shows no correlation between bradycardia and the analyzed variables.

**3. Risk of Bias assessment**

**Table** Domain-specific risk of bias evaluation according to the RoB2 framework.

| **Domain** | **Low** | **Some concern** | **High** |
| --- | --- | --- | --- |
| Bias arising from the randomization process | 28 (100%) | 0 (0%) | 0 (0%) |
| Bias due to deviations from intended interventions | 19 (67.9%) | 2 (7.1%) | 7 (25%) |
| Bias due to missing outcome data | 13 (46.4%) | 3 (10.7%) | 12 (42.9%) |
| Bias in measurement of the outcome | 25 (89.3%) | 1 (3.6%) | 2 (7.1%) |
| Bias in selection of the reported result | 9 (32.1%) | 3 (10.7%) | 16 (57.1%) |
| Overall | 9 (32.1%) | 4 (14.3%) | 15 (53.6%) |

**Figure** Risk relationship across individual domains evaluated using the RoB2 framework.


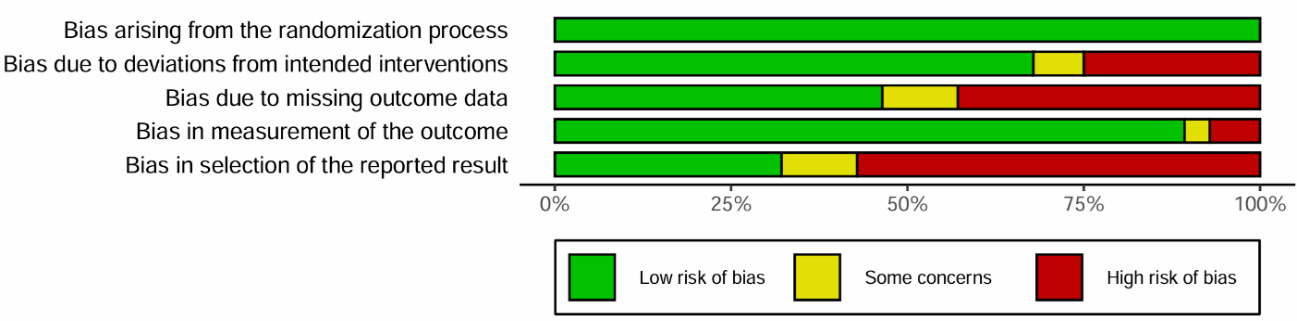


**4. GRADE presentation**

**Table** GRADE classification for main outcomes.

| **Outcome (Setting / Subgroup)** | **Nº of Studies (Participants)** | **Pooled Estimate (95% CI)** | **Certainty (GRADE)** | **Rationale for Rating** |
| --- | --- | --- | --- | --- |
| Procedural Success (Dose 2‒3 mcg.kg^-1^; Low/Mod RoB) | 5 (286) | 84.04% (79.21–87.91) | High | No serious risk of bias, inconsistency, or imprecision. |
| Sedation Onset Time (Overall) | 34 (1609) | 18.9 min (16.6–21.4) | Very Low | Downgraded 2 levels for very serious inconsistency (I² = 99%) and 1 level for high risk of bias. |
| Sedation Duration Time (Overall) | 28 (1368) | 60.3 min (52.7–69.1) | Low | Downgraded 2 levels for very serious inconsistency (I² = 99.3%). |
| Desaturation (Low/Mod RoB) | 14 (846) | 3.07% (1.90–4.92) | Moderate | Downgraded 1 level for imprecision (wide CI relative to effect). |
| Hypotension (SBP < 20% basal; Low/Mod RoB) | 4 (359) | 6.94% (4.68–10.20) | Moderate | Downgraded 1 level for imprecision (wide CI relative to effect). |
| Bradycardia (Low/Mod RoB) | 9 (629) | 4.78% (1.97–11.12) | Very Low | Downgraded 2 levels for serious inconsistency (I² = 73%) and 1 level for serious imprecision. |
